# Mid-Infrared Sensing and Ultrafast Photoresponse in Silicon-Based Plasmonic Detectors

**DOI:** 10.1021/acsphotonics.5c02857

**Published:** 2026-05-20

**Authors:** Mauro David, Alicja Dabrowska, Masiar Sistani, Zehao Song, Francesco Maraspini, Yina Wu, Matthaeus Wenk, Andreas Fuchsberger, Lilian Vogl, Peter Schweizer, Rolf Szedlak, Benedikt Schwarz, Walter Michael Weber, Gottfried Strasser, F. Javier García de Abajo, Juraj Darmo, Bernhard Lendl, Alois Lugstein

**Affiliations:** † Institute of Solid State Electronics, Technische Universität Wien, Gußhausstraße 25-25a, 1040 Vienna, Austria; ‡ Institute of Chemical Technologies and Analytics, 27259 Technische Universität Wien, Getreidemarkt 9, 1060 Vienna, Austria; § ICFO-Institut de Ciencies Fotoniques, The Barcelona Institute of Science and Technology, Castelldefels, 08860 Barcelona, Spain; ∥ Structure and Nano-/ Micromechanics of Materials, 28272 Max Planck Institute for Sustainable Materials, Max-Planck-Straße 1, 40237 Düsseldorf, Germany; ⊥ ICREA-Institucio Catalana de Recerca i Estudis Avançats, Passeig Lluis Companys 23, 08010 Barcelona, Spain; # Institute of Photonics, Technische Universität Wien, Gußhausstraße 27, 1040 Vienna, Austria

**Keywords:** mid-infrared sensing, photodetector, CMOS-compatible, silicon photonics, plasmonics, integrated optics

## Abstract

Mid-infrared photonics enables a large number of applications in chemical sensing, medical diagnostics, environmental monitoring, and optical communications, but widespread adoption of this technology is hindered by the lack of compact, CMOS-compatible photodetectors capable of room-temperature operation. This work presents an Al–Si–Al planar heterostructure acting as a plasmonic photodetector that leverages an electrostatically tunable Schottky barrier to detect sub-bandgap mid-infrared photons (5–7 μm) in silicon with a responsivity ∼0.9 mA/W, <0.4 mA/cm^2^ dark current density, and a broad and uniform spectral response that does not require cooling. Fast internal dynamics with time constants of 3.7 ps and 1.4 ns are observed at 1560 nm, suggesting the potential for high-speed operation. Our monolithic and crystalline Al–Si heterostructure features abrupt interfaces obtained without epitaxy, ensuring CMOS compatibility and scalability. We demonstrate the potential of this device for mid-infrared sensing by detecting and spectrally characterizing water molecule absorption lines. By leveraging plasmonically generated hot-carriers combined with the inherent scalability of silicon-based technology, our work opens new pathways for cost-effective and high-speed infrared photodetectors suitable for next-generation integrated photonic systems.

## Graphical abstract

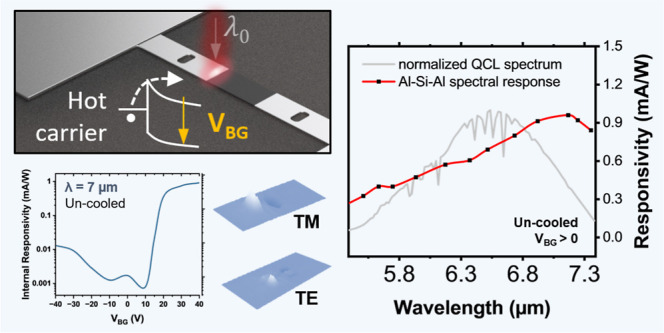

## Introduction

The rapid evolution of photonics over the last decades has been a critical driver in advancing optical communication technologies, pushing high-speed data transfer,^1^ and enabling groundbreaking applications across multiple fields. Among the most promising developments is mid-infrared (mid-IR) photonics, which holds potential for chemical sensing,^2,3^ medical diagnostics,^4^ astronomy,^5^ security screening,^6,7^ and free-space optical communication^8–10^ among other feats.

The mid-IR spectral region, spanning wavelengths from 2.5 to 25 μm, is particularly valuable due to its unique molecular fingerprinting capabilities, enabling the detection of a wide range of chemical and biological substances.^2,11^ However, the widespread adoption of mid-IR photonics has been hindered by the lack of cost-effective, scalable, and CMOS-compatible photodetectors, which are crucial for integration into compact systems and mass production.^12^ An ideal mid-IR detector should offer high responsivity, low noise, fast response times, and a broad, uniform spectral response, while operating at room temperature, maintaining a compact footprint, and remaining cost-effective.^13^ Nonetheless, achieving all these characteristics simultaneously remains a significant challenge.

Current commercial solutions rely primarily on thermal and direct-photon detectors. Thermal detectors,^14–16^ while capable of broadband operation across a wide spectral range (2–2000 μm), suffer from inherently slow response times in the millisecond regime due to their reliance on heat accumulation and dissipation.^17^ In contrast, photon detectors, such as those based on mercury–cadmium–telluride (MCT), provide significantly faster response times (on the order of nanoseconds) and higher sensitivity, although with narrowband operation, yet tunable spectral coverage in the mid-IR. However, these advantages come at the cost of complex fabrication processes, high power consumption, and the need of cooling to suppress dark currents.^18,19^ Moreover, material toxicity concerns are leading to regulatory restrictions on mercury and cadmium in Europe,^20^ limiting the widespread adoption and future scalability. Alternative III–V semiconductor-based detectors, including multiquantum well superlattices^21–27^ and quantum dots,^28–31^ have been explored, but their integration with silicon photonics remains challenging,^32^ limiting scalability and cost-effectiveness. Recent advances in van der Waals heterostructures^33–36^ and 2D materials-based photodetectors^37,38^ have demonstrated promising performance across the infrared, but currently face limitations in scalability and operation speed, often with response times exceeding 10 ns. To overcome these limitations, recent efforts have focused on extending silicon photonic functionality^32^ into the mid-IR by using CMOS-compatible materials such as Ge^39,40^ and SiGe alloys.^41,42^ Defect-mediated absorption mechanisms^43,44^ and GeSn-based photodetectors^45,46^ have demonstrated the ability to enable room-temperature, CMOS-compatible, high-speed detection (up to 15 GHz) but are limited to wavelengths below 2.5 μm.

An alternative promising approach is plasmonic detection,^47,48^ where nonradiative decay of surface plasmons at metal–semiconductor interfaces generates hot carriers on femtosecond time scales.^49–52^ These hot carriers can be harnessed to enable sub-bandgap photodetection, particularly when coupled with resonant plasmonic structures.^53^ Although CMOS-compatible plasmonic photodetectors have been reported for wavelengths up to 5.3 μm,^52,54–57^ their extension to longer wavelengths remains largely unexplored. Building on our previous studies of Al–Ge–Al nanowire devices,^52^ the present work introduces a top-down fabricated Al–Si–Al plasmonic photodetector that employs an electrically tunable Schottky barrier. For the first time, we demonstrate room-temperature sub-bandgap photodetection in silicon extending into the mid-IR range up to 7 μm, achieving an internal responsivity of ∼0.9 mA/W without the need for any cooling system. Our device features a low dark-current density (<0.4 mA/cm^2^), a broad and uniform spectral response, and enhanced hot-electron current generation via back-gate voltage tuning. Owing to its plasmonic detection mechanism, the device intrinsically supports ultrafast operation, with projected response times down to 3.4 ps upon further optimization. Moreover, the fabrication process results in a monolithic and crystalline Al–Si–Al heterostructure with abrupt interfaces,^58^ eliminating the need for epitaxial growth. Importantly, this device successfully detects roto-vibrational absorption lines of atmospheric water vapor, underscoring its potential for practical mid-IR sensing applications. By leveraging CMOS-compatible materials and fabrication processes, our work paves the way for fast, scalable, and cost-effective mid-IR detectors, offering significant advantages in sensing, imaging, and optical communication applications.

## Results and Discussion

### Device Structure

Figure 1a,b show the schematic and microscope image of a fully featured Al–Si–Al device fabricated using a CMOS-compatible thermal-driven Al–Si exchange process,^58^ following a methodology described in refs 59 and 60 (see Experimental Section for more details). The realization of the devices started with a silicon-on-insulator (SOI) wafer with a 100 nm thin and slightly p-doped (∼10^16^ cm^–3^) device layer that was patterned into microribbons of 25 × 2.5 μm^2^ using laser lithography and reactive ion etching. After device passivation and Al contact definition, an Al–Si exchange reaction at 773 K was induced via rapid thermal annealing, causing Al to diffuse from the lithographically defined Al contact pads into the Si ribbon.^59,61^ The extreme mismatch in the diffusion coefficients of Al and Si,^58^ and the absence of an intermetallic phase, leads to sharp interfaces and a crystalline Al–Si–Al heterostructure, as shown in Figure 1f. Moreover, this process determines the Si channel length (∼5 μm), which can be precisely controlled by adjusting the annealing duration.

**Figure 1:**
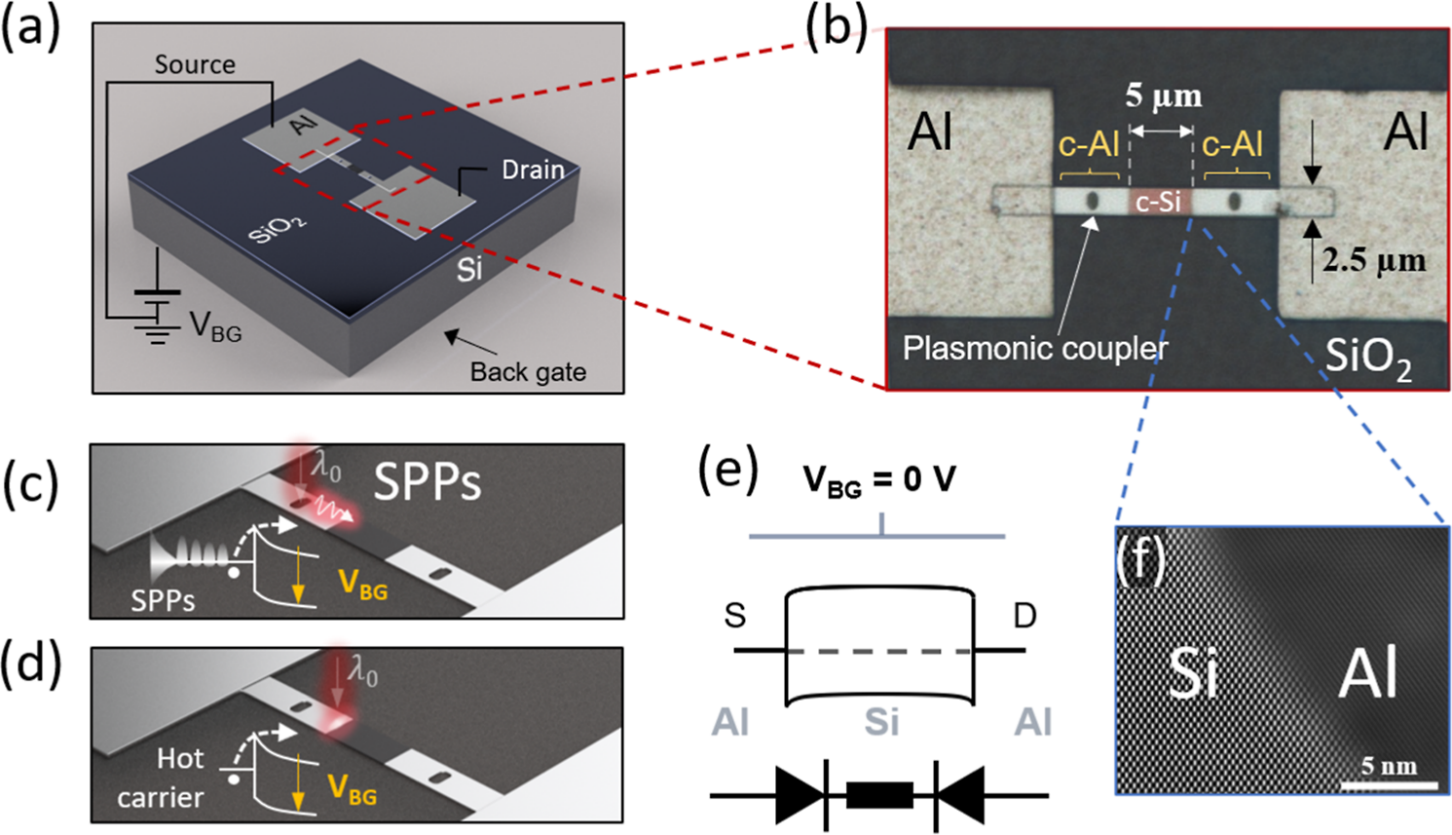
Overview of our Al–Si–Al photodetector. (a) Schematic illustration of the Al–Si–Al photodetector. (b) Microscope image showing a top view of the device. The Si core section (*L*_Si_ = 5 μm, *w*_Si_ = 2.5 μm) is flanked by two crystalline Al (c-Al) leads, including two oval-shaped trenches acting as simple plasmonic couplers. The roughness difference between the sputtered Al region and the c-Al section obtained through the thermal exchange reaction is clearly visible. (c) Schematic representation of light illumination at the plasmonic coupler, where surface plasmon polaritons are generated and propagate toward the Al–Si interface. Inset: Hot-electron extraction at the metal–semiconductor interface, detected by lowering the barrier after applying a back-gate voltage *V*_BG_. (d) Schematic of direct excitation at the interface, with the inset illustrating the hot-carrier detection mechanism. (e) Band diagram of the Al–Si–Al heterostructure device for a slightly p-doped Si channel at *V*_BG_ = 0. The lower section presents an equivalent circuit of the device, which consists of two back-to-back Schottky diodes. (f) High-resolution scanning transmission electron microscopy images of the Si–Al interface, highlighting the crystalline nature of the fabricated heterostructure.

Finally, the sample was mounted on a copper plate to enable contact with the handle wafer, which serves as the global back-gate. Thus, the device resembles a back-gated Schottky barrier field-effect transistor (SB-FET), with the SOI wafer’s buried oxide layer acting as the gate dielectric. Figure 1e shows the band diagram when no back-gate voltage *V*_BG_ is applied, along with its equivalent electronic circuit.

As previously shown,^60^ the monolithic Al leads in this configuration can function both as electrical feed lines and as waveguides for surface-plasmon polaritons (SPPs). SPPs can be excited when a normally incident transverse magnetic polarized light couples into the crystalline Al lead either directly near the edge with the Si section or via interaction with the indentations carved in the metal (see Figure 1b,c and electromagnetic simulations in Supporting Information). SPPs generated upon illumination of a distant metal surface feature can propagate to the Al–Si interface. The optical fields of both these propagating plasmons and light directly incident at the Al–Si interface are amplified by the sharp metal edge, where they can decay and generate hot electrons. Hot electrons originating from the excitation of states near the Fermi level of Al possess energies that can either overcome or tunnel through the Schottky barrier and be injected into the semiconductor. The injection efficiency is governed by the electron’s potential landscape across the Al–Si junction, the electronic and structural properties of the Schottky barrier, and the orientation of electrons’ wave vector relative to the Al–Si interface. However, we note that photocurrent mapping indicates that a higher photocurrent is generated when light is coupled directly at the metal–semiconductor junction. This is in agreement with our calculations for illumination of the oval-shaped coupler, which produces a weak field at the interface compared with direct irradiation (see Supporting Information). This is because the nonradiative decay of plasmons excited at the metallic edge^62^ occurs in direct proximity to the semiconductor section, minimizing losses associated with SPP propagation along the waveguide. It should be noted that optimizing the coupling conditions by creating a dedicated coupler for SPP excitation could further enhance the overall device response.^63,64^

### Electrical Characterization

The carrier transport mechanism in the heterostructure under bias follows the characteristic behavior of an SB-FET. Figure 2a presents the *I*–*V* curves of a device with a channel length of *L*_Si_ = 5 μm at various positive back-gate voltages, while Figure 2b shows the respective transfer characteristics. The drain-source current (*I*_DS_) exhibits an ambipolar transistor behavior as a function of the back-gate voltage, with *I*_DS_ increasing for both positive and negative gate voltages. Figure 2c shows the band diagrams at points 1 and 2 (marked in Figure 2b), illustrating the origin of the observed ambipolar behavior and the evolution of the band structure under varying bias conditions. At the metal–semiconductor interface, a thermionic emission current is always present, while the tunneling current becomes significant only when the gate-induced band bending reduces the effective barrier heights and width, enhancing hot electron transmission. Due to the global back gate, the applied gate voltage symmetrically modulates the Schottky barriers at both Al–Si contacts. A negative gate voltage induces an upward band bending of the silicon channel, increasing the Schottky barrier height for electrons, while simultaneously lowering the barrier for holes and enhancing hole conduction. In contrast, a positive gate voltage induces downward band bending, reducing the effective barrier for electrons and enhancing electron injection into the Si channel, while blocking the hole-based current. In terms of photodetector functionality, it is expected that hot carriers generated via nonradiative plasmon decay facilitate hot-electron detection at *V*_BG_ above ∼0 V, whereas for voltages below this threshold, the resulting band alignment enhances the tunneling probability for hot holes. This behavior provides significant versatility, allowing for precise tuning of the detection mechanism to optimize performance across a specified wavelength range or for specific detection wavelengths.

**Figure 2:**
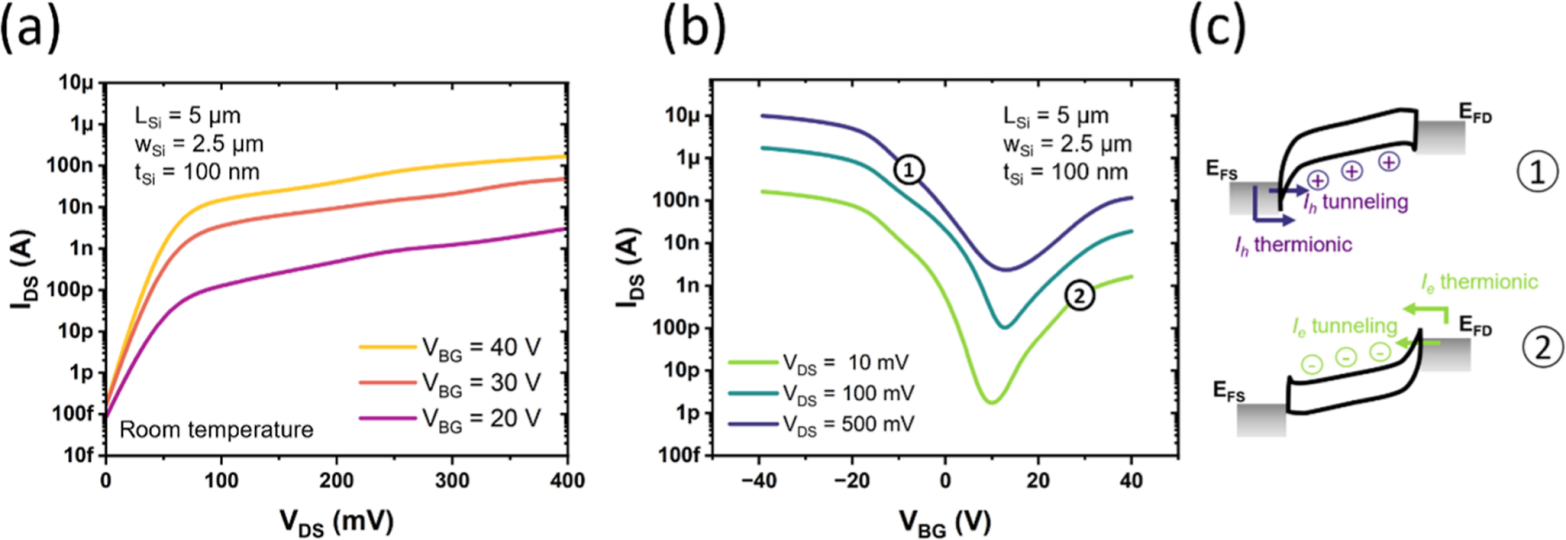
Electrical characterization of the Al–Si–Al heterostructure. (a) Output characteristics of an Al–Si–Al SB-FET with a Si channel length of *L*_Si_ = 5 μm, width *w*_Si_ = 2.5 μm and thickness *t*_Si_ = 100 nm at different back-gate voltages. (b) Transfer characteristics of the same device, measured at various drain biases (*V*_DS_). (c) Band diagrams at points 1 and 2 explaining carrier transport under different *V*_BG_ biases (not to scale).

### Electro-Optical Characterization

A common interband silicon detector typically responds to wavelengths between 400 and 1100 nm, given by the bandgap of Si, which is approximately 1.1 eV, corresponding to a cutoff wavelength of 1100 nm. For wavelengths exceeding the silicon bandgap threshold, photons lack sufficient energy to generate electron–hole pairs, rendering this semiconductor ineffective in the infrared range. Here, we demonstrate that this fundamental limitation can be effectively overcome by engineering and electrically tuning the Schottky junction, enabling the detection of wavelengths well beyond the silicon bandgap and extending into the mid-infrared regime, all under uncooled operating conditions. Figure 3a,b illustrate the transport mechanism of the actual device for sub-bandgap detection in photovoltaic operation mode (*V*_DS_ = 0 V) when light illuminates the metallic structures and excites plasmons in the vicinity of the Al–Si interface. In Al, surface plasmon decay results in hot electrons and holes with a continuous energy distribution, primarily driven by interband transitions near the W point.^65^ Without any external gate bias (*V*_BG_ = 0), only hot carriers with sufficient energy to overcome the Schottky barrier height (Φ_SB_) contribute to the photoinduced current, making the device function as a typical photoemissive detector.^55,66^ Given the slight p-type doping of Si in the device under study, the bands are essentially flat.^67^ Under these conditions, due to the Fermi level pinning approximately at the midgap point,^58,67–72^ the detection wavelength threshold extends to around 2.1 μm, corresponding to an effective barrier height of approximately 600 meV.^55^ By applying a positive gate bias, the effective barrier height (Φ_B_) can be reduced, facilitating electron tunneling through the barrier and injection into the Si channel, and resulting in a measurable plasmon-induced current.

**Figure 3:**
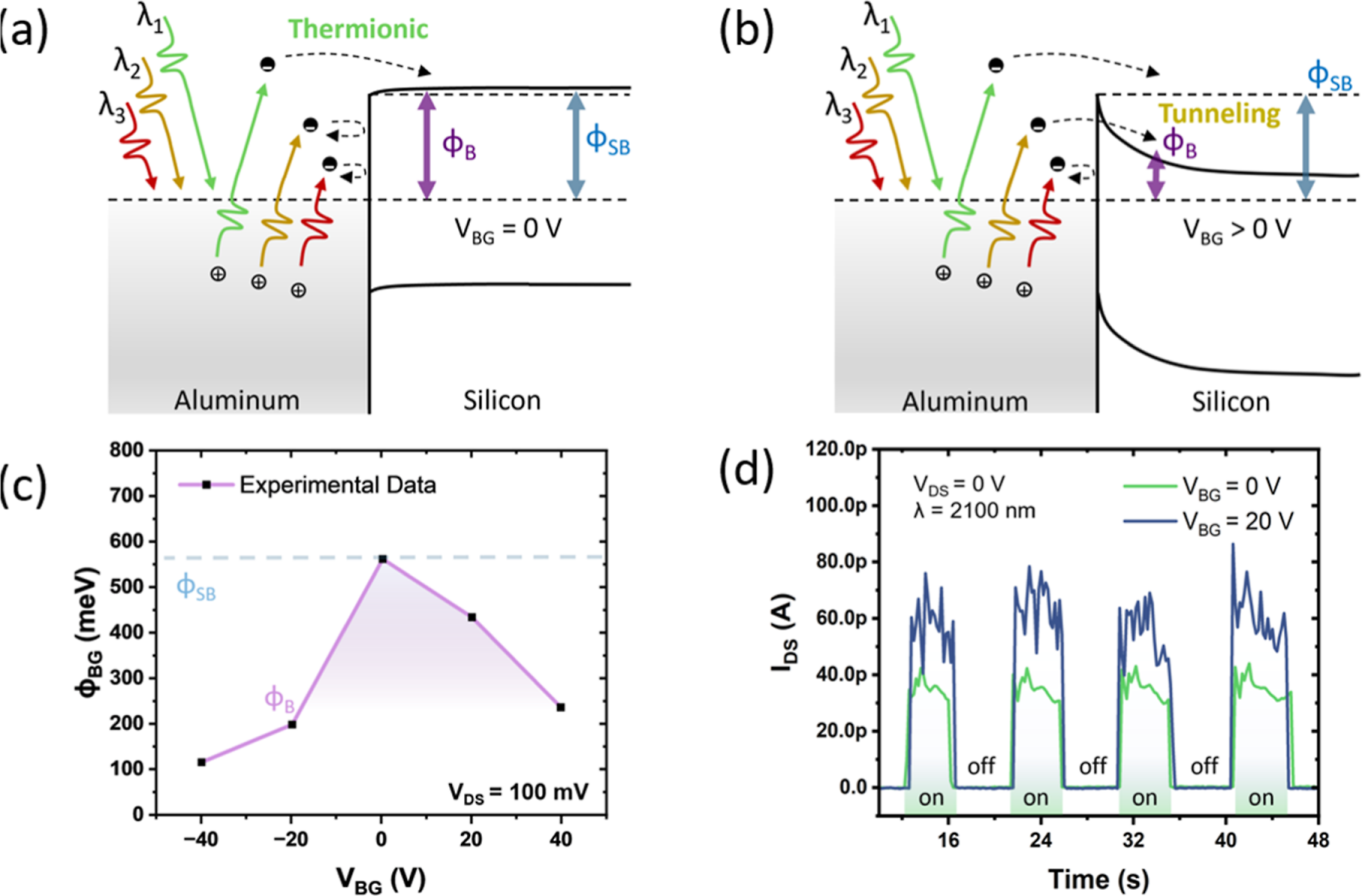
Illustration of the device working mechanism. (a) The metal–semiconductor junction under zero bias, and (b) the effect of applying a positive gate bias. The green, yellow, and red wavy arrows represent light with high, medium, and low photon energies. As the barrier is reduced, hot carriers with lower energies can tunnel through the barrier, enabling the detection of sub-bandgap radiation. (c) Extracted effective barrier height (Φ_B_) at different back-gate voltages, determined using the Richardson method at *V*_DS_ = 100 mV. (d) Time-resolved drain current at two different gate voltages under illumination with 2100 nm light wavelength. The “on” and “off” labels indicate when the laser is switched on and off, providing a clear reference for the illumination cycle.

A similar argument applies to holes under negative gate bias. In this case, band bending happens in the opposite direction, reducing the effective barrier height for hole injection, which is consistent with the behavior observed in the transfer characteristics.

To quantify the effective Schottky barrier height, temperature-dependent transfer measurements were performed at various back-gate voltages and analyzed using the Richardson method,^73^ with the extracted values plotted in Figure 3c. From this data, we obtain a maximum barrier height of ∼550 meV around *V*_BG_ = 0. This value is close to half the Si bandgap (*E*_g_ = 1.12 eV), confirming Fermi level pinning near the middle of the gap. At back-gate voltages of +40 V and −40 V, the effective barrier height reduces to 240 and 120 meV, respectively, suggesting the potential for mid-IR detection up to wavelengths of 12 μm. To initially validate this assumption, the devices were tested under near-IR excitation using a SuperK Extreme laser source (NKT Photonics). The light was focused onto the Al–Si interface (as shown in Figure 1d) using a microscope objective, and plasmon-induced photocurrent was detected up to 2100 nm, well beyond the Si bandgap limit of 1100 nm, confirming the proposed working principle. Figure 3d shows the drain-source current in the time domain under pulsed 2100 nm illumination at two different back-gate voltages, notably, without any biasing (i.e., *V*_DS_ = 0). At *V*_BG_ = 0, the plasmon-induced peak current averages around 38 pA, while increasing the gate voltage to *V*_BG_ = 20 V enhances the photocurrent to 60 pA, demonstrating a back-gate-dependent photoresponse. Interestingly, at *V*_BG_ = 0, the current retains the same polarity as when *V*_BG_ is positive, suggesting that the electron current remains more favorable even at zero back-gate voltage. Additionally, the noise level increases with back-gate voltage, significantly surpassing the dark current noise, which is approximately 50 fA. When the back-gate voltage is biased negatively, the current polarity reverses, indicating a shift in carrier transport (holes). At this wavelength, negative back-gate voltages also lead to a slight deterioration in the noise performance, resulting in a reduced signal-to-noise ratio. This can be attributed to the increased dark current noise, as suggested by the transfer characteristics shown in Figure 2b, where hole transport is typically higher by 1-to-2 orders of magnitude than the electron current (see the Experimental Section for further details about the noise). This suggests that electron transport becomes more favorable for the plasmon-induced photocurrent generation at longer wavelengths, well beyond the bandgap of silicon.

To confirm the plasmonic origin of the photocurrent and exclude alternative mechanisms such as photothermal effects, we investigated the polarization dependence of the device response at an illumination wavelength of 1.3 μm. Figure 4 illustrates the response of an Al–Si–Al device (*V*_DS_ = 0, *V*_BG_ = 20 V) under two orthogonal polarization conditions: transverse magnetic (TM) and transverse electric (TE). The data in this figure clearly demonstrates a polarization dependence of the photocurrent upon illumination. Specifically, the response is uniform at the interface under TM polarization (Figure 4a,b,e, suggesting a homogeneous distribution of plasmonic coupling at the interface), while under TE polarization (electric field parallel to the interface, see Figure 4d,c,f) two distinct peaks appear at the metal edge with a weaker peak current. This strong polarization-dependent behavior provides compelling evidence of the field enhancement, and, consequently, the working principle of our proposed detector (see Supporting Information for simulations of the local field enhancement for different polarizations). It should be noted that, under our experimental conditions, achieving perfectly pure TE or TM polarization states was not possible. Therefore, when we refer to TM polarization, we indicate that approximately 96% of the light is TM-polarized, with a residual 4% of the electric field intensity oriented in the orthogonal (TE) direction. Nevertheless, the clear polarization-dependent response strongly supports our interpretation of plasmonic excitation as the dominant mechanism. In fact, if alternative mechanisms such as photothermal heating were dominant, no polarization dependence would be expected.

**Figure 4:**
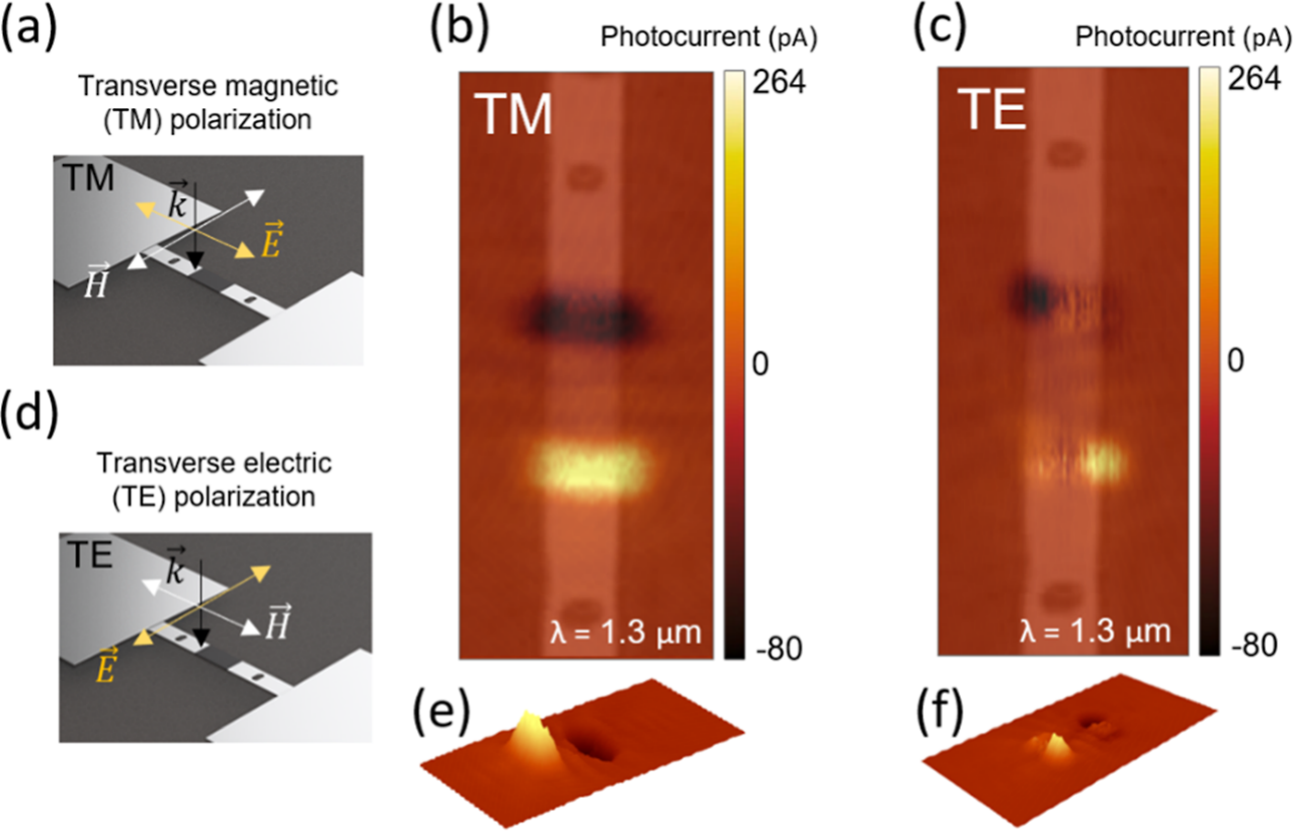
Photocurrent maps of an Al–Si–Al device with a 5 μm-long Si section, and couplers positioned 3.5 μm away from the interfaces. Panels (a,d) show polarization schemes employed in the experimental setup for TE and TM polarization, respectively; (b,c) show the respective measured maps for TM and TE polarization, along with a superimposed transparent microscope image of the device, and (e,f) show the respective 3D maps for TM and TE polarization.

Finally, it is important to note that all measurements presented here were conducted at room temperature, without any cooling system, and at *V*_DS_ = 0. Cooling the devices could potentially improve the noise characteristics, leading to enhanced performance.

To evaluate the electro-optical response in the mid-IR range, the devices are tested using a mid-IR setup, schematically shown in Figure 5a. In this configuration, light from a tunable quantum cascade laser (QCL) is coupled into the detector via a Cassegrain reflecting objective. The QCL operates in pulsed mode with a repetition rate of 100 kHz, a duty cycle of 5%, and a maximum current of 1020 mA (average optical power of 20.2 mW at a wavelength of 6.52 μm). The back-gate of the device is controlled using a Keithley SourceMeter, and the current is measured with a Zurich Instrument lock-in amplifier.

**Figure 5:**
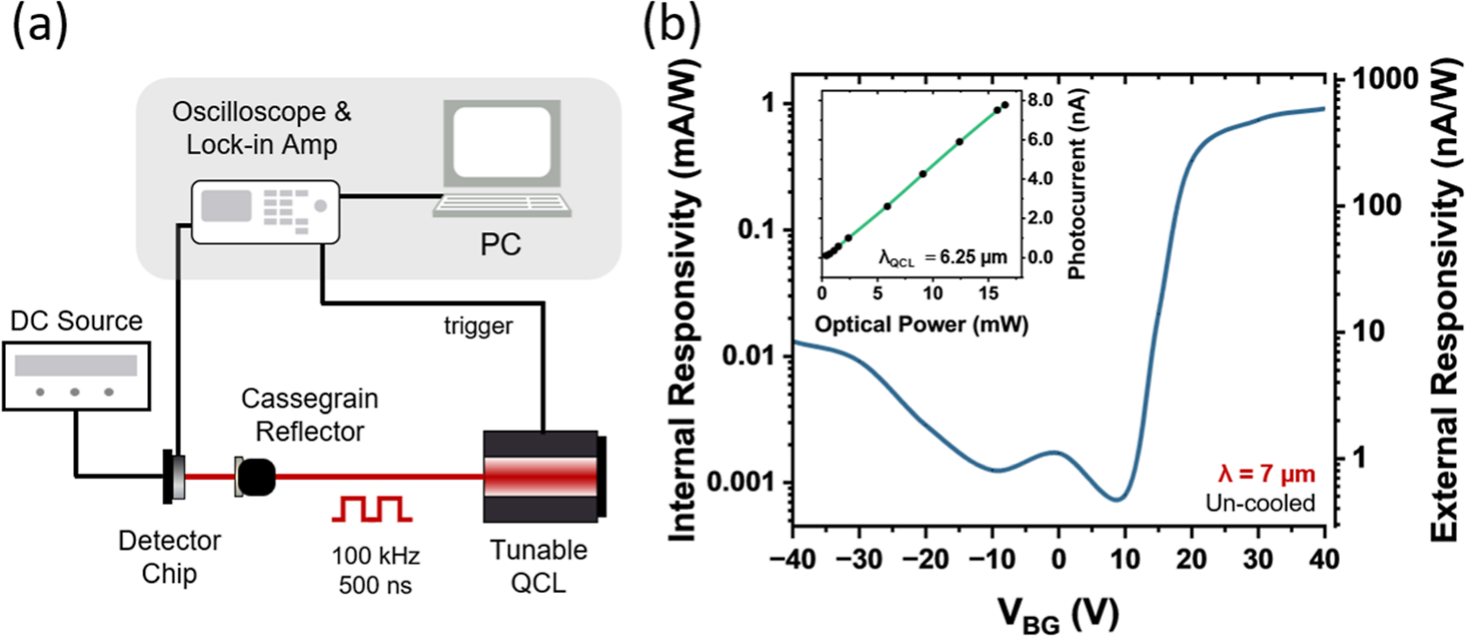
Mid-IR measurement setup and device performance. (a) Schematic of the mid-IR measurement setup, which includes a tunable QCL source, with light focused onto the detector using a Cassegrain reflecting objective. The back-gate bias is controlled by a DC source, and the photoinduced current is measured using a lock-in amplifier. (b) Responsivity as a function of back-gate voltage, demonstrating the device’s operational principle. Inset: Photocurrent as a function of incident light power at 6.25 μm with *V*_BG_ = 40 V, demonstrating a linear response regime.

The sensitivity of the device is quantified by estimating external and internal responsivity. The external responsivity (*R*_ext_) quantifies the photocurrent generated by illumination relative to the total laser power incident on the detector chip. However, since the device is significantly smaller than the laser beam area after focusing by the Cassegrain reflector (the beam diameter is approximately 22 μm), a substantial portion of the optical energy impinges outside the active area of the detector. To account for this mismatch, the coupling efficiency is typically estimated by evaluating the spatial overlap between the focused beam spot size and the detector’s active area (see Experimental Section for more details). The device’s optical area is approximated by its electrical cross-section (consistent with how Schottky detectors are often treated) for the purpose of calculating the internal responsivity (*R*_int_). Therefore, *R*_ext_ is defined as *R*_ext_ = *I*_ph_/*P*_QCL_, where *I*_ph_ is the photocurrent collected by the device and *P*_QCL_ is the optical input power, measured using a power meter at the output of the Cassegrain reflector. The internal responsivity is defined as *R*_int_ = *I*_ph_/*P*_coupled_, where *P*_coupled_ is the optical power measured after the reflector, scaled by the ratio of the device’s active region to the focused beam area. Figure 5b shows the measured responsivity at 7 μm as a function of back-gate voltage. Remarkably, despite the relatively low probability of hot electron tunneling across the barrier, the detector achieves responsivities of up to 0.912 mA/W at a back-gate voltage of 40 V, with the responsivity increasing monotonically above *V*_BG_ = 10 V. A significantly lower responsivity is observed for negative back-gate bias (i.e., hole injection), presumably due to differences in tunneling probabilities and effective barrier heights between holes and electrons, which is consistent with the measurements performed with the near-IR setup at 2.1 μm, and confirmed by the extracted specific detectivity (D*) values vs back-gate voltage (see Experimental Section for more details). When the responsivity reaches its maximum (*V*_BG_ = 40 V), the device shows an NEP of 53.0 pW and a detectivity D* of 9.3 × 10^7^ Jones, while the maximum detectivity of ∼2 × 10^8^ Jones is obtained at *V*_BG_ = 20.

It should be noted that although the applied gate voltages may appear relatively high, this result primarily originates from the thick oxide layer used in the present prototype (∼400 nm). In practical implementations, employing thinner oxides would proportionally reduce the required voltages to 1–2 V, without affecting the device’s operational principles.^74,75^

Detector linearity is also an essential requirement for accurate measurements. The inset of Figure 5b shows the measured photocurrent as a function of the incident light power, measured at 6.25 μm with *V*_BG_ = 40 V and demonstrating a clear linear relationship that confirms that the device can withstand high laser power densities (up to 165 kW/cm^2^ in pulsed operation) with no saturation. This linearity is particularly important for spectroscopic applications, where a precise power dependence is critical for reliable measurements.

Although the responsivity reported here might seem modest, these results demonstrate a performance comparable to other room-temperature, CMOS-compatible photodetectors for similar wavelength ranges.^40,76–78^ To place these results in context, Table 1 compares our results with a summary of representative CMOS-compatible mid-infrared photodetectors reported in the literature within comparable spectral ranges.

**Table 1:** Comparison of Representative Room Temperature CMOS-Compatible Mid-infrared Detectors

detection mechanism	material system	operating wavelength (μm)	peak responsivity (mA/W)	D* (Jones)	cutoff frequency (MHz)	device footprint (μm^2^)
pyroelectric mid-infrared detector^76^	AlN	4.17	109	9.4 × 10^6^	–	2.5 × 105
strained-relaxed GeSn membranes^77^	GeSn	1.5–4.0	∼1	–	–	400
waveguide-integrated bolometric detector^40^	Ge/TiO_2_/Ti/TiO_2_	4.18	0.037	–	0.1	32
plasmonic polarizers integrated with CMOS microbolometers^78^	Al/SiO_2_/Al	7–11	∼5	∼3 × 10^9^	–	0.032
electrically tunable plasmonic detector (this work)	Al/Si	5–7	∼1	∼1 × 10^8^	115	0.25

Other promising technologies rely on waveguide-integrated mid-IR photodetectors based on 2D materials, such as graphene,^79,80^ black phosphorus,^81^ and WS_2_/HfS_2_ photodetectors.^82^ While they offer broad spectral coverage, they often suffer from limitations in dark current or absorption efficiency. Moreover, their reliance on exfoliation or transfer-based fabrication severely limits scalability and compatibility with wafer-scale, high-volume manufacturing.

To conclude this section, we want to highlight that further development of the presented Al–Si–Al device could enable substantially higher performance. In fact, in the current configuration, due to the large beam diameter, the illumination inevitably covers both interfaces, leading to the measured signal reflecting overlapping contributions from currents generated at each interface. Introducing design asymmetry and selectively masking one of the interfaces could significantly enhance the device’s response by minimizing these overlapping effects. Additionally, increasing the device footprint, cooling the device, and biasing the two aluminum contacts (*V*_DS_) could further improve the device response to infrared photons. Overall, we believe that the presented work establishes a strong foundation for further exploration and optimization, with clear opportunities for significant performance enhancement. Even more important, the CMOS-compatible fabrication process and the current architecture, based on Si and Ge, well-established low-loss materials for mid-IR waveguides, opens a direct route toward much-needed monolithic integration with passive components. Bridging the gap between Si or Ge waveguides and on-chip detection provides a scalable and cost-effective platform, positioning this technology for broad adoption in high-volume and advanced applications.^12^

### Mid-IR Spectroscopic Measurements

Building on the fundamental understanding of the device’s operational mechanism along with baseline electrical and optical characterization, we aim to demonstrate its performance and applicability for mid-IR sensing under ambient conditions and zero-bias operation. Figure 6 presents a qualitative spectral measurement of roto-vibrational transitions of water vapor.

**Figure 6:**
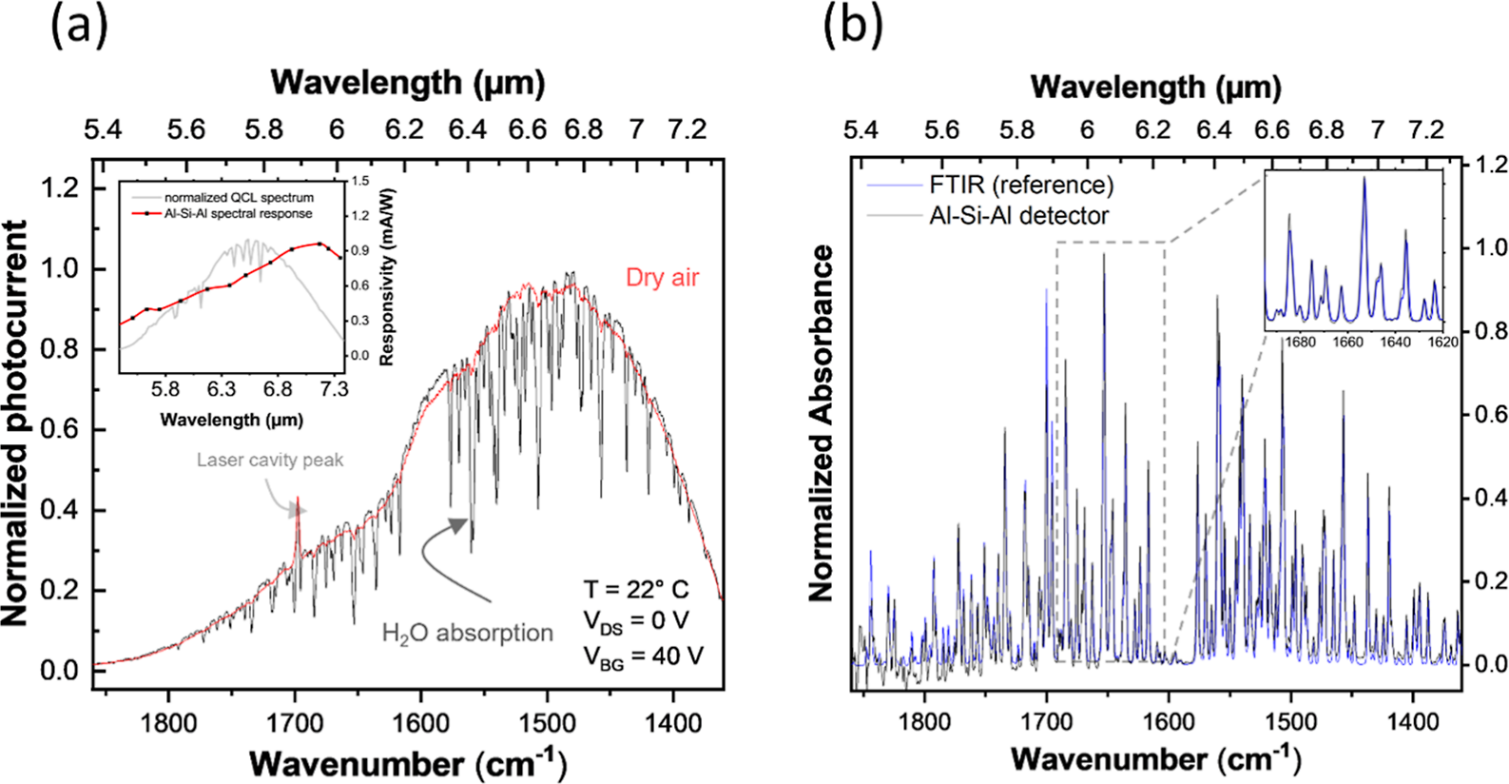
Mid-IR spectral response and water vapor detection with the Al–Si–Al photodetector. (a) Spectral measurement of water vapor using the mid-IR detector. The red line represents the detector’s photocurrent when the setup is well-purged with dry air, while the black line corresponds to measurements taken with environmental air, showing the typical strong absorption feature of water vapor in the range between 5.4 and 7.3 μm. Inset: Comparison of the QCL spectrum measured with a power meter and the responsivity of the Al–Si–Al detector as a function of wavelength, highlighting the uniform spectral response of the presented device. (b) Comparison of the measurement taken with the detector (black curve) and a conventional FTIR spectrum (blue curve), highlighting the agreement between the two methods. Inset: Zoomed-in view of the region between 1690 and 1620 cm^–1^.

In Figure 6a, the red curve corresponds to the detector’s response when the mid-IR setup of Figure 5a is encapsulated in a chamber and well purged with dry air, while the black curve represents the response under normal environmental conditions (temperature of 22 °C, relative humidity of ∼45%), where water vapor absorption is observed. Additionally, the registered spectrum intensity profile shows good agreement with the emission profile of the mid-IR QCL (shown in gray in the inset of Figure 6a) measured with a power-meter, indicating that the detector exhibits a relatively flat and uniform response in this wavelength region (red line in the inset of Figure 6a). This is further corroborated in Figure 6b, which compares the absorbance obtained from the detector under mid-IR QCL excitation (black curve) with a reference spectrum acquired using a conventional FTIR system (blue curve), demonstrating strong agreement between the two measurement techniques. These results validate the accuracy and reliability of our detector for spectroscopic applications, confirming its potential for real-world use in mid-IR sensing. It should be noted that the peak observed at 1700 cm^–1^ is inherent to the laser cavity and consistently appears in all measurements, even when using different detectors. Therefore, this peak is not related to the actual detector device.

### Intrinsic Response Dynamics

The hot-carrier mechanism underlying our detection mechanism is expected to be intrinsically fast (i.e., commensurate with the few-fs lifetimes of such carriers). In fact, it is well-known that photodetectors based on Schottky diodes can reach high-speed operation of tens of GHz.^83,84^ To validate this intuition, we investigate the intrinsic response dynamics of the device. However, evaluating the device’s signal bandwidth in the mid-IR range is challenging due to the lack of efficient high-speed modulators in this wavelength range. Therefore, we performed measurements at 1560 nm, as we believe this wavelength choice does not compromise the generality of the results, and is consistent with the approach adopted in previous studies.^41^ To gain insight into the time scale of the internal processes that generate the device output signal, we have applied the pump–probe technique.^85^ We illuminated the device with 90 fs-long pulses produced by the mode-locked fiber laser and measured the corresponding response of the device. Namely, each laser pulse is split into two pulses of equal energy with a varying time delay between them before they arrive at the device. If the device is fast enough, it can respond to each of the two pulses equally. In contrast, if the second (probe) pulse strikes the device while it is still responding to the first (pump) pulse, the device cannot respond equally to both pulses. We have plotted this response difference in Figure 7a. More details on the used pump–probe technique applied to the photodetector can be found in ref 86.

**Figure 7:**
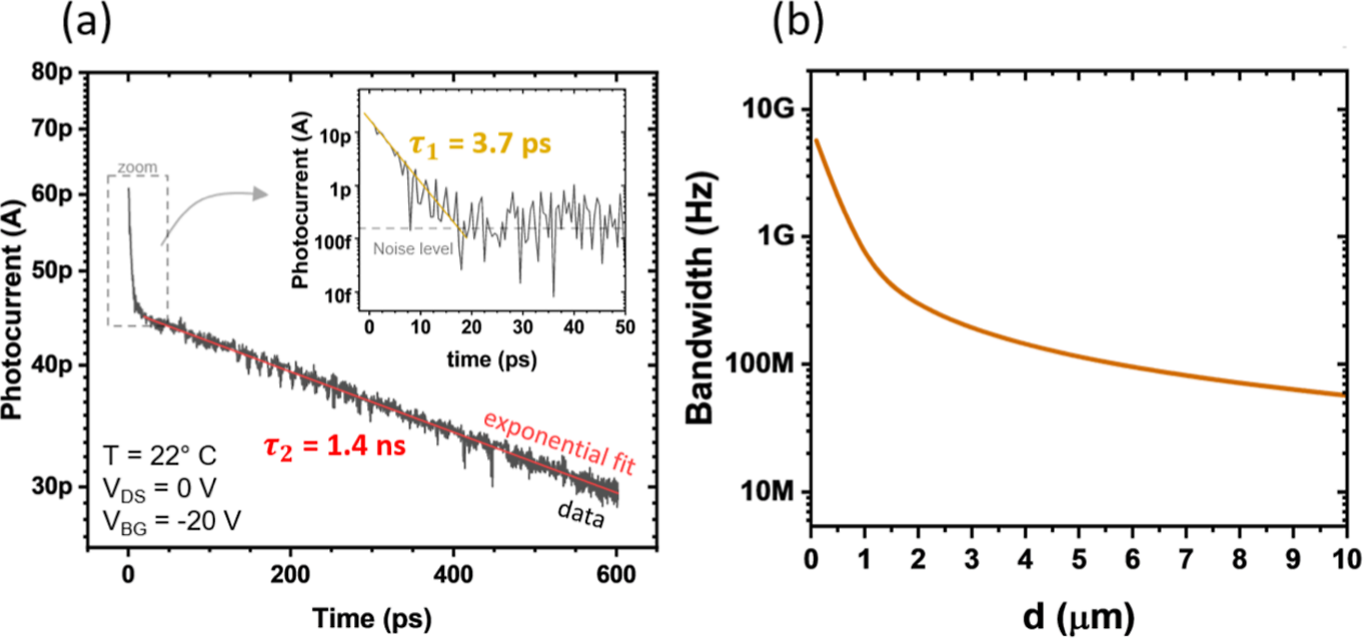
(a) Differential photocurrent induced by a 90 fs-long 1560 nm light probe pulse for different time delays after the pump pulse strikes the device. Two regions are identified with distinctly different time constants corresponding to different mechanisms of photocurrent generation. Inset: Differential photocurrent after removing the slow component. (b) Calculated transit time-limited bandwidth as a function of the Si segment length when used in photovoltaic mode.

We have found that the detector signal in the time domain consists of two main components with distinctly different time constants: τ_1_ = 3.7 ± 0.6 ps, attributed to hot-carrier injection from the Al contact, and τ_2_ = 1.4 ± 0.15 ns, associated with the carrier transport in the Si section. The relative contributions to the total detector signal are 29% and 71%, respectively. These results indicate that the practical speed of the device (*f*_cutoff_ ≈ 114 MHz) is currently limited by electron transport in the silicon channel of the current device architecture. It follows that to enhance the device speed, reducing the silicon channel length,^86,87^ could help minimize this limitation. In fact, the transit-time-limited bandwidth can then be estimated using the following expression:^73^

$$f_{t}=\frac{\sqrt{2}v}{\pi d}$$

where *v* is the velocity of carriers in Si (extracted from the pump and probe measurements, leading to ∼1.1 × 10^5^ cm/s) and d is the distance between the two aluminum electrodes. Figure 7b shows the expected 3 dB cutoff for different segment lengths, highlighting the potential for such a device to reach above GHz operation for short segment lengths. Biasing the heterostructure (i.e., *V*_DS_ ≠ 0 V) is expected to increase the operational speed, albeit at the expense of higher noise.

To place our results in context, while this speed is still below the record values reported for quantum-well infrared photodetectors (QWIPs, >70 GHz),^24^ or quantum cascade detectors (QCDs, ∼25 GHz^88,89^ ), it already compares favorably to several commercial mid-IR photodetectors (e.g., MCTs ∼ 1 GHz,^90^ interband cascade infrared photodetectors ∼7 GHz,^91^ and a resonant-cavity infrared detector ∼6 GHz^92^) and is significantly superior to thermal, pyroelectric, and thermopile detectors.

The detector’s external responsivity at 1560 nm to the 90 fs-long pulses is about 1.3 μA/W (or 1.3 fC/nJ of the pulse), which is comparable to the external responsivity observed for mid-IR photons.

## Conclusion

In this work, we report a monolithic, CMOS-compatible Al–Si–Al heterostructure photodetector, leveraging an electrically tunable Schottky barrier to achieve mid-IR detection at room temperature. This approach extends the detection capabilities of silicon-based devices into the mid-IR range, particularly between 5 and 7 μm, with an internal responsivity of ∼0.9 mA/W (NEP of 53.0 pW and a D* of 9.3 × 10^7^ Jones) under ambient, uncooled operation, thereby overcoming the intrinsic absorption limitations of silicon. The detector, operated in a photovoltaic regime, exhibits a low dark-current density (<0.4 mA/cm^2^) and a rather flat spectral responsivity. The temporal response of the actual detector is primarily limited by carrier transport within the silicon channel, characterized by a slower time constant of τ_2_ = 1.4 ns. Reducing the channel length and installing the device in a high-frequency RF package would enable modulation frequencies exceeding 10 GHz. This, along with the successful detection of water absorption lines, demonstrates the device’s potential for mid-IR applications. Additionally, their monolithic architecture, compact footprint, and broadband detection capability make our detectors well-suited for integration into Si and Ge photonic waveguides and multispectral imaging systems. These results hint at the potential of these devices for next-generation mid-IR silicon photonic detectors compatible with CMOS technology, enabling new possibilities in sensing, imaging, and telecommunication technologies.

## Experimental Section

### Device Fabrication

A commercially available SOI wafer with a (100)-oriented, 100 nm-thick, lightly p-type (boron-doped) device layer (resistivity of 1–10 Ω·cm, corresponding to a doping concentration on the order of 10^16^ cm^–3^), 381 nm-thick buried oxide (BOX), and a 500 μm-thick handle wafer was used as the starting material. The wafer was diced into 11 mm × 8 mm pieces. To remove the native SiO_2_ layer, the samples were immersed for 10 s in buffered oxide etch (BOE 7:1 VLSI, from MicroChemicals), followed by rinsing in water and drying with N_2_. Further surface cleaning was performed by ultrasonication in acetone for 5 min to improve adhesion between the sample and the photoresist. The samples were spin-coated with AZ5214E photoresist at 6000 rpm for 40 s, followed by a soft bake at 100 °C for 60 s. The microribbon structures, each featuring two oval-shaped cutouts, were defined using laser lithography. The exposed regions were etched using reactive ion etching (Oxford PlasmaPro 100 Cobra ICP Etching System) with gas flows of 50 sccm SF_6_ and 10 sccm O_2_ at 35 °C for 150 s. After etching, the samples were cleaned in acetone and isopropanol, followed by oxygen plasma treatment (300 W for 180 s) to remove residual photoresist. A 12 nm-thick Al_2_O_3_ passivation layer was deposited onto the patterned samples using atomic layer deposition (ALD) at 200 °C. Aluminum contact pads were patterned using laser lithography. Before metal deposition, the samples were immersed in BHF (7:1) for 30 s to remove both the Al_2_O_3_ passivation layer and the native SiO_2_ in the contact regions. To prevent reoxidation, the samples were immediately transferred to a sputtering chamber, where a 125 nm-thick Al layer was deposited via sputtering. The excess metal was removed using a liftoff process in acetone at 55 °C for 1 h. To induce the Al–Si exchange reaction, the samples underwent rapid thermal annealing at 773 K in a forming-gas atmosphere (90% N_2_/10% H_2_). The total annealing duration was approximately 30 min to ensure complete Al–Si exchange in the designated regions. To achieve precise control over the Si channel length, the process was typically carried out in 5 min cycles, with the Si channel monitored under an optical microscope after each cycle. This procedure allowed careful observation of Al diffusion, minimizing overdiffusion and ensuring reproducible device performance. The previously etched oval-shaped trenches in the Si microribbons transformed into recessed regions within the crystalline Al leads. For electrical measurements, the samples were mounted onto a copper plate using silver paste. A wire was then contacted to the copper plate, enabling the use of the SOI wafer’s handle layer as a global back-gate via the 381 nm-thick buried oxide layer.

### Electrical Characterization

The electrical measurements shown in Figure 2 were carried out using a Karl Suss manual probe station in combination with a Keysight 4156B precision semiconductor parameter analyzer. To extract the Schottky barrier height (Φ_B_) of the SB-FET shown in Figure 3c, temperature-dependent electrical measurements were carried out using a probe station (Lake shore Cryotronics) with a thermos chuck and an integrated temperature controller. Needle probes were employed to apply a bias to the drain and source electrodes, as well as the Cu slide, which served as the back-gate contact. Measurements were conducted over a temperature range of 320 to 400 K, with a 10 min stabilization period to ensure thermal equilibrium before data acquisition. The Schottky barrier height was determined using the Richardson plot method, a widely recognized technique for analyzing thermionic emission behavior.^73^

### Optical Characterization

The devices were characterized using three measurement setups:

The first (near-IR) setup was used to extract basic electrical characteristics and evaluate the device’s response to illumination at a wavelength of 2100 nm. Broadband white light from a SuperK Extreme (NKT) laser source was directed into a monochromator (SuperK Select, NKT). The monochromatic output was generated using an acoustic-optical tunable filter (AOTF) system, which selected wavelengths in the infrared range (1100–2100 nm). The filtered light was then coupled into a WITec Alpha300 system, passed through a 50:50 beam splitter, and focused onto the device using a Zeiss 100× objective. Calibrated power meter measurements yielded ∼3.2 μW at 1800 nm, with a decreasing trend toward longer wavelengths; accordingly, the incident optical power at 2100 nm is estimated to be on the order of ∼1 μW or lower. The time-domain measurements of Figure 3d were performed using a Keysight 1500A semiconductor parameter analyzer in transient mode. The reported traces correspond to raw photocurrent signals acquired without lock-in amplification, with an integration time of 80 μs and a sampling interval of 100 ms.

The second (mid-IR) setup was designed to investigate the device’s mid-IR detection capabilities. A tunable external cavity QCL from DRS Daylight Solutions, operating within a wavelength range of 5.4–7.3 μm, served as the illumination source. For most of the experiments, the laser emitted 500 ns pulses at a 100 kHz repetition rate. The beam was focused onto the detector using a 36× Cassegrain reflective objective. A Keithley SourceMeter was used to apply a bias to the device gate, while the photocurrent was measured with an MFLI lock-in amplifier from Zurich Instruments. A power meter was used to measure the optical power after the Cassegrain reflector for responsivity calculations.

The third setup was used for pump–probe measurements,^85^ employing 90 fs pulses at 1560 nm from a femtosecond laser (Menlo System F9). The pulse train was split into pump and probe beams, modulated at distinct but phase-locked frequencies using a dual-section optical chopper, and recombined before being focused onto the Al–Si–Al device via a microscope objective. A motorized delay stage controlled the time delay between pulses, and the differential photocurrent was extracted from the detector using a lock-in amplifier. The effective time resolution at the sample was ∼560 fs, limited by dispersion in the optical path. More details about the method and setup can be found in ref 86.

### Figures of Merit

In this work, the detectors are based on Al–Si–Al heterostructures, illuminated perpendicularly from the top. In this configuration, defining the active area used for extracting the detector’s figures of merit is not straightforward. Here, we used the device’s electrical cross-section (i.e., the product of its width and thickness, *A*_el_ = *w* × *t* = 0.25 μm^2^) as only hot carriers at the metal/Si interface contribute to the plasmon-induced photocurrent. In fact, as shown in Figure S2 of the Supporting Information, the optical field intensity (relevant for creating hot carriers that contribute to the current in the device) is peaked at the Al–Si interface under normal illumination (an effect due to plasmonic enhancement by the response of the sharp metal edge at the interface). Then, the field decays dramatically within a few tens of nanometers away from the interface, so only the part of the field closer to it can contribute to the current. In addition, hot carriers produced by photon absorption of Al electrons decay away from their excitation region with a finite inelastic mean free path (IMFP), such that Al regions farther apart from the interface than such an IMFP do not contribute to the current.^93^ Moreover, this is consistent with how Schottky detectors are often treated.

As shown in Figure 5b the photocurrent response of the actual device depends critically on the applied back-gate voltage. When the laser illuminates the detector at a wavelength of λ_0_ = 7 μm, at a V_BG_ = 40 V bias, we obtain a maximum photocurrent of approximately *I*_ph_ = 8 nA at a laser power of 13.7 mW measured with a power meter after the Cassegrain reflector. The beam spot size at the focal point was estimated using the following equation:^94^

$$2w_{0}=\frac{4\lambda_{0}f}{\pi D}$$

where 2*w*_0_ is the beam diameter at focus, *f* is the focal length of the lens, and *D* is the beam diameter before the lens. Using an infrared camera, we recorded the beam’s Gaussian profile before the focusing lens. From this, we extracted a 1/*e* ^2^ waist of 2*w*_0_ = 22.38 μm, corresponding to a circular beam area at focus of 393.5 μm^2^. The incident power on the detector, *P*_inc_, is then given by the fraction of the total optical power spatially overlapping with the detector’s active area:

$$P_{inc}=P_{beam}\cdot M_{0}=P_{beam}\cdot \frac{A_{\det}}{A_{bf}}$$

where *A*_bf_ is the area of the circular beam at focus, defined by its 1/*e* ^2^ waist. It follows that the device responsivity at 7 μm (and *V*_BG_ = 40 V) is 0.9125 mA/W.

In this work, the detectors were operated in photovoltaic mode (i.e., zero bias across the device), thus rendering Johnson–Nyquist noise the predominant noise contribution. The only externally applied voltage was the back-gate bias (*V*_BG_), used to modulate the effective height of the Schottky barrier at the Al–Si interface. As demonstrated in several previous studies,^95^ this modulation is directly reflected in the overall device resistance. Consequently, and in line with established practice in the literature, we defined the noise from the shunt resistance, which we extracted from the device’s dark *I*–*V* characteristics measured in the ±10 mV range.^96^ Nevertheless, it should be noted, that the reported detectivity corresponds to a Johnson–Nyquist noise-limited estimate and therefore represents an intrinsic upper bound of the device performance. Figure 8a reports the extracted resistance values as a function of *V*_BG_, along with the corresponding rms noise currents, calculated as

$$i_{n,\mathrm{rms}}=\sqrt{\frac{4k_{B}T\Delta f}{R_{sh}}}$$

where *k*_B_ is the Boltzmann constant, *T* the temperature, and *R*_sh_ the shunt resistance.

**Figure 8:**
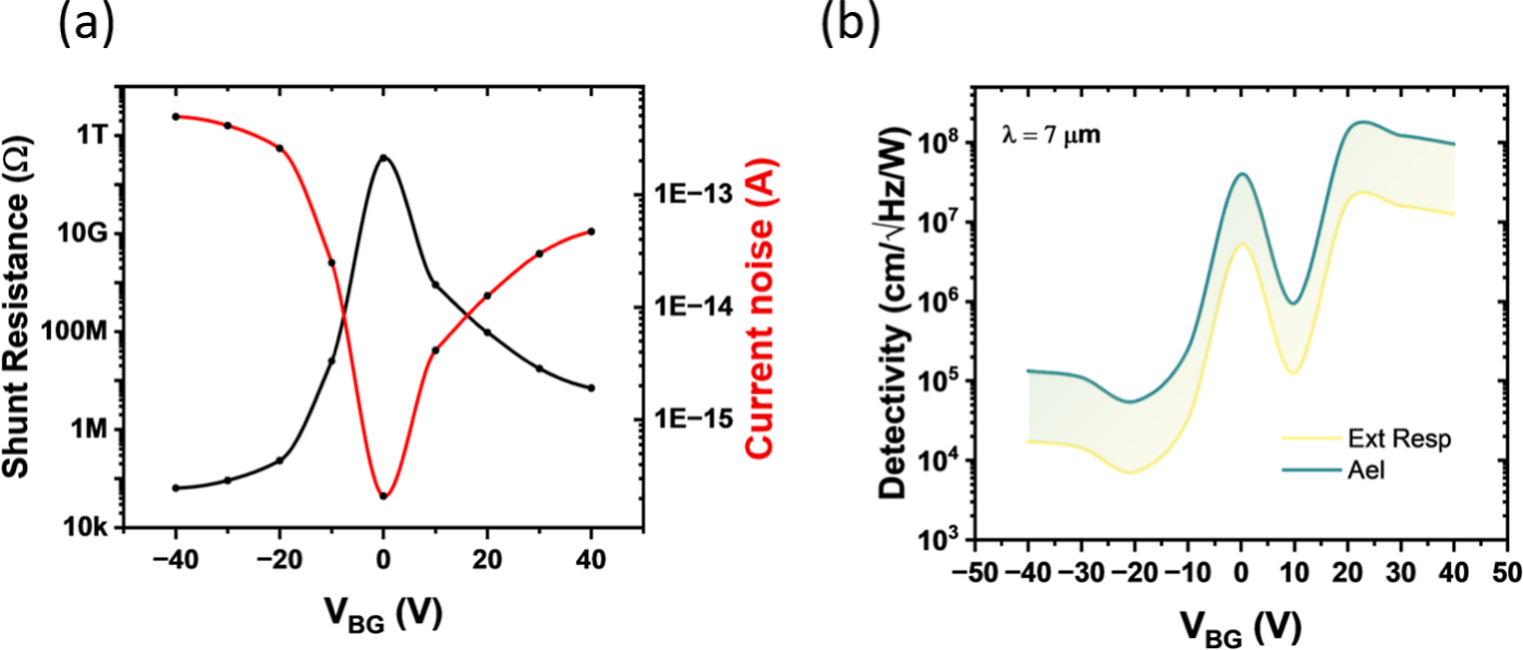
(a) Extraction of the device shunt resistance (black curve) for different applied back-gate voltages (*V*_BG_) at room temperature (295 K) and corresponding Johnson–Nyquist noise values derived from the extracted resistances (in red). (b) Calculated detectivity at 7 μm for the Al–Si–Al device as a function of*V*_BG_. The green curve corresponds to calculations based on the electrical area used as active area (as for the calculation of *R*_int_), while the yellow curve uses the external responsivity for the derivations of D*. The shaded region indicates the difference arising from the active-area definition.

The overall performance of a photodetector is commonly benchmarked using the specific detectivity (D*), which is obtained by normalizing the NEP to the detector active area and the measurement bandwidth Δ*f*:

$$D^{*}=\frac{\sqrt{A_{\det}\Delta f}}{NEP}$$

The bandwidth can be computed from the integration time constant:

$$\Delta f=\frac{1}{2t_{c}}$$

As our measurements were performed using a Zurich Instrument Lock-in with a cutoff frequency of 1337 Hz, according to the manual,^97^ this corresponds to a time constant of ∼52 μs, yielding a value of D* of 9.3 × 10^7^ Jones at *V*_BG_ = 40 V. It should be noted that in the extraction of the above figures of merit, due to uncertainties in precisely defining the detector’s effective optical area, the calculated quantities presented here may vary depending on the choice for the definition of the area. Table 2 summarizes the range of values obtained under two extreme assumptions: the estimated active area, and the scenario in which only the external responsivity is considered.

**Table 2:** Comparison of Detector Figures of Merit Based on Different Definitions of the Effective Optical Area at *V*_BG_ = 40 V

area (μm^2^)	responsivity (μA/W)	NEP (pW)	D* (Jones)
*A*_el_ = 0.25	915	53	9.3 × 10^7^
–(external)	0.58	82,680	1.2 × 10^7^

As observed in Table 2, while the responsivity and NEP vary significantly with the selection of the area, the D* values remain largely consistent.

Figure 8b shows the calculated D* values for these two extreme cases as a function of back gate voltages *V*_BG_ under mid-infrared light illumination at 7 μm. In the mid-IR range, D* increases for positive back-gate voltages, where the detector favors hot-electron transport over hole transport, consistent with the discussion above regarding the higher noise observed at negative voltages when measuring in the near-infrared region. Around zero back-gate bias, the detectivity also appears relatively high. However, this value is somewhat misleading, as it is influenced by the naturally low noise level of the device under zero-bias conditions. For this reason, in the discussion above, we chose to emphasize the responsivity, which provides a more qualitative and practical indication of the device performance under operational conditions.

### Spectroscopic Measurements

For spectroscopic measurements, the experimental setup was identical to the mid-IR characterization system described above, with the key modification that all components were enclosed in a sealed chamber and purged with dry air to minimize humidity and associated absorption effects. The mid-IR QCL was operated in pulsed mode, with a repetition rate of 100 kHz, 5% duty cycle, and a current of 1020 mA. The Al–Si–Al heterostructure detector was biased at 40 V on the back-gate to maximize the device’s responsivity. After the initial measurements in a low-humidity environment, the chamber was vented to allow ambient water vapor to re-enter. To improve signal accuracy and reduce noise, each measurement was performed over 10 scans, and the results were averaged to reduce random noise contributions.

## Supplementary Material



## Data Availability

The data that support the findings of this study are available on request from the corresponding author A.L.

## Notes

Estimate of the efficiency of light-plasmon electron tunneling based on an effective-potential theory; formulation of the tunneling-current model; electromagnetic calculations for the illuminated aluminum–silicon interface (PDF)
