# Supplementary material for: Mid-Infrared Sensing and Ultrafast Photoresponse in Silicon-Based Plasmonic Detectors
Source: ACS Photonics. 2026 May 20;13(11):3026–39. doi: 10.1021/acsphotonics.5c02857 (PMC13237816; doi:10.1021/acsphotonics.5c02857)
Supplement: Supplementary file 1 [file ph5c02857_si_001.pdf]

Mid-Infrared Sensing and Ultrafast Photoresponse in Silicon-Based Plasmonic Detectors

Mauro David, Alicja Dabrowska, Masiar Sistani, Zehao Song, Francesco Maraspini, Yina Wu, Matthaeus Wenk, Andreas Fuchsberger, Lilian Vogl, Peter Schweizer, Rolf Szedlak, Benedikt Schwarz, Walter Michael Weber, Gottfried Strasser, F. Javier García de Abajo, Juraj Darmo, Bernhard Lendl, and Alois Lugstein

We present an estimate of the efficiency for light-plasmon electron tunneling in our structures (Sec. S3) based on a self-contained effective-potential theory (Sec. S2) combined with electromagnetic calculations for an illuminated aluminum-silicon interface (Sec. S1). The theory incorporates a phenomenological description of tunneling electrons in the one-dimensional potential associated with the aluminum-silicon interface, which enters through the corresponding electron Green function (Appendix A) and initial (unperturbed) conduction electrons in aluminum (Appendix B). This theory is formulated to the linear order in the light-electron interaction.

S1. NEAR-FIELD IN AN ALUMINUM-SILICON WAVEGUIDE INTERFACE

In Fig. S1, we present calculations for the near field near an aluminum-silicon interface based on a finite-element method (COMSOL). We assume plane-wave illumination normal to the plane of representation, with incident light polarization as indicated by the white arrows. A top view of the structure is outlined by black lines, showing a waveguide of 2.5 μm in width (along y) and consisting of aluminum and silicon to the left and right of a central interface. The waveguide has a height of 100 nm (along z) and is supported on silica. We plot the electric field intensity for the component along x (parallel to the waveguide), which is expected to dominate the electron tunneling process. The plane of representation is the top surface of the waveguide.

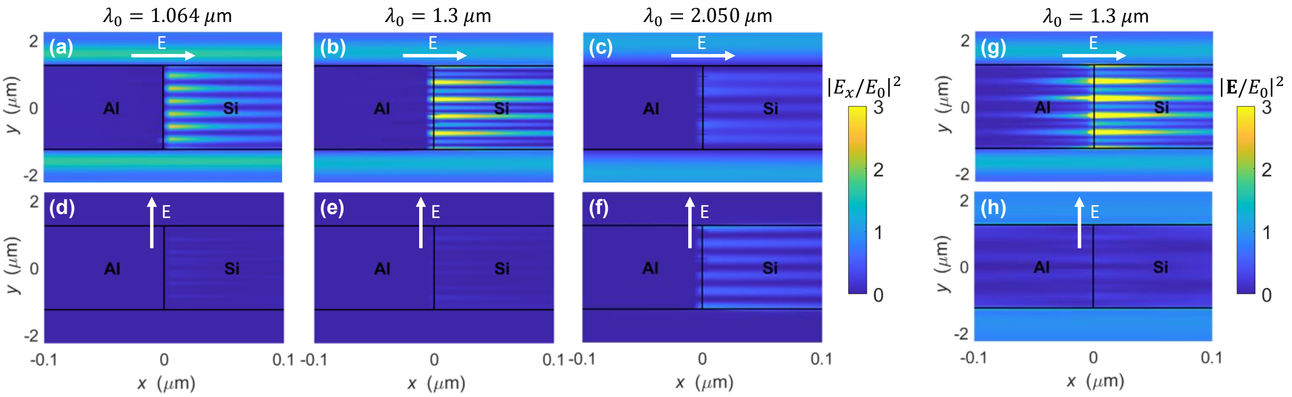

FIG. S1: **Near electric field in an aluminum–silicon waveguide junction upon direct external illumination. (a-f)** Intensity of the electric field component along x (the waveguide direction) normalized to the incident field E_0 for illumination by a plane wave with polarization as indicated by the white arrows, which are parallel and perpendicular to the waveguide in the top (a-c) and bottom (d-f) plots, respectively. The intensity is represented at the top surface of the waveguide, whose width and height are 2.5 μm and 100 nm along y and z, respectively. The black lines outline the boundaries of the waveguide materials. We consider different light wavelengths of 1064 nm (a,d), 1.300 nm (b,e), and 2050 nm (c,f). **(g,h)** Same as (b,e), but showing the intensity contributed by all field components.

For polarization across the interface (Fig. S1a-c), we observe a substantial coupling near the interface, which is associated with plasmon launching to the left, mainly mediated by the x component of the electric field acting on the interface, as well as transverse standing waves on the silicon side. The latter are characterized by a spacing of roughly half the light wavelength in silicon (i.e., they originate in Fabry-Perot-like interference across the width of the silicon waveguide) and undergo plasmonic enhancement at the interface with the metal. Accordingly, the fringe spacing increases with light wavelength.

In contrast, for polarization parallel to the interface (Fig. S1d-f), the x component of the field only takes significant values on the silicon side, also due to coupling to standing waves across the waveguide. On the metal side, plasmonic standing waves are launched at the lateral sides, giving rise to standing waves with polarization along y (Fig. S1h), and thus, the x field component becomes very weak.

In summary, for light incidence with polarization across the aluminum-silicon interface, the near electric field develops a substantial x component, undergoing plasmonic enhancement at the metal edge. For polarization parallel to the interface, we expect a much weaker electron tunneling current because the x component of the field is also substantially smaller.

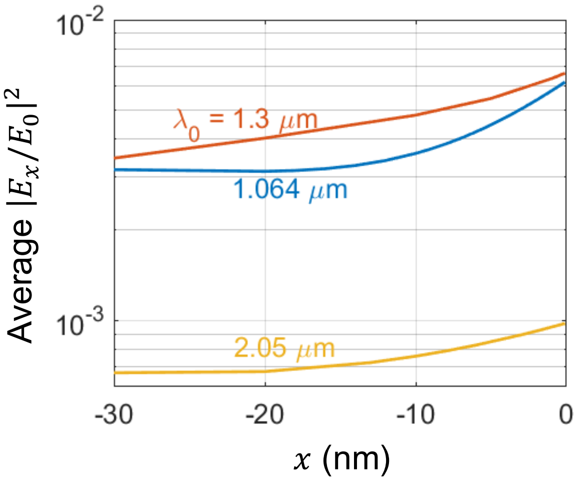

FIG. S2: **Decay of the exciting optical field in aluminum away from the interface.** We represent the normalized intensity $|E_x/E_0|^2$ of the x electric field component normalized over the cross section of the aluminum waveguide as a function of distance x from the interface.

In Fig. S1a-c, we observe a sharp decay of the E_x field in aluminum away from the interface. This is quantified in Fig. S2 by representing the average intensity of the x electric field component (i.e., $A^{-1}\int_A dydz|E_x/E_0|^2$, where the integral runs over the cross section A of the aluminum waveguide and is normalized to its area) as a function of x. The decay length increases with wavelengths, and in particular, for a wavelength of 1.3 μm, the field is reduced by a factor of two at a distance of ~ 20 nm.

In Fig. S3, we explore the coupling of infrared light to guided plasmons in a waveguide similar to the aluminum part of Fig. S1. We perforate the waveguide with an elliptical hole and calculate the near electric field and Poynting vector at two different light wavelengths within selected planes, as indicated in the sketch of Fig. S3a. The induced near field is dominated by a beaming effect produced by scattering on either side of the hole, with a magnitude comparable to the external field. In contrast, the field induced far from the hole has a much lower magnitude, already indicating a rather poor coupling efficiency. When plotting the Poynting vector component parallel to the waveguide direction x on the perpendicular $y-z$ plane at a certain distance from the hole (central and right panels in Fig. S3d,e), we observe the effect of energy propagation due to scattering by the hole (i.e., the Poynting vector along x should vanish in the absence of the hole because no energy is transported along that direction upon illumination by a normally incident plane wave, so only the hole-scattered field contributes to this component of the Poynting vector). We observe that energy is mainly transported near the lateral edges of the waveguide, similar to the propagation of microwaves in microstrip technology [1]. Upon integration of the Poynting vector maps over $y-z$ and normalization to the incident light intensity, we obtain the coupling cross section (i.e., the effective area over which the incident plane wave energy reaches the corresponding transverse plane in the form of plasmons). Such a cross section decays with the distance from the hole (Fig. S3b) due in part to inelastic attenuation during plasmon propagation. In addition, the cross section exhibits a non-monotonic dependence on light wavelength (Fig. S3c), characterized by a broad spectral peak associated with the coupling to loosely bound plasmon resonances in the metallic hole [2].

S2. THEORY OF PLASMON-ASSISTED TUNNELING

We describe electron tunneling based on the potential energy landscape shown in Fig. S4. For simplicity, independent electrons are considered, ruled by the approximate Hamiltonian

$$\hat{\mathcal{H}}_0(\mathbf{r}) = -\frac{\hbar^2\nabla^2}{2m_e} + U(x),$$

where the electron mass is assumed to be uniform, isotropic, and equal to the free-electron mass m_e. This is a good approximation for aluminum, while for silicon we neglect the complexity of the band structure, including the fact that it features six conduction band minima along $(0,0,\pm1)$ and symmetry-related directions, featuring anisotropic effective masses that include a longitudinal one close to m_e [along $(0,0,\pm1)$].

We assume monochromatic plasmons with an associated optical electric field $\mathbf{E}(\mathbf{r},t) = \mathbf{E}(\mathbf{r})\,e^{-i\omega t} + \text{c.c.}$ of frequency ω. Each of the conduction electrons in aluminum is studied separately, starting from an unperturbed wave function (see Appendix B)

$$\psi^0_{\mathbf{Q},n}(\mathbf{r},t) = A^{-1/2} e^{i\mathbf{Q}\cdot\mathbf{R}}\,\varphi^0_n(x)\,e^{-i(\varepsilon_n+\varepsilon_Q)t}, \tag{S1}$$

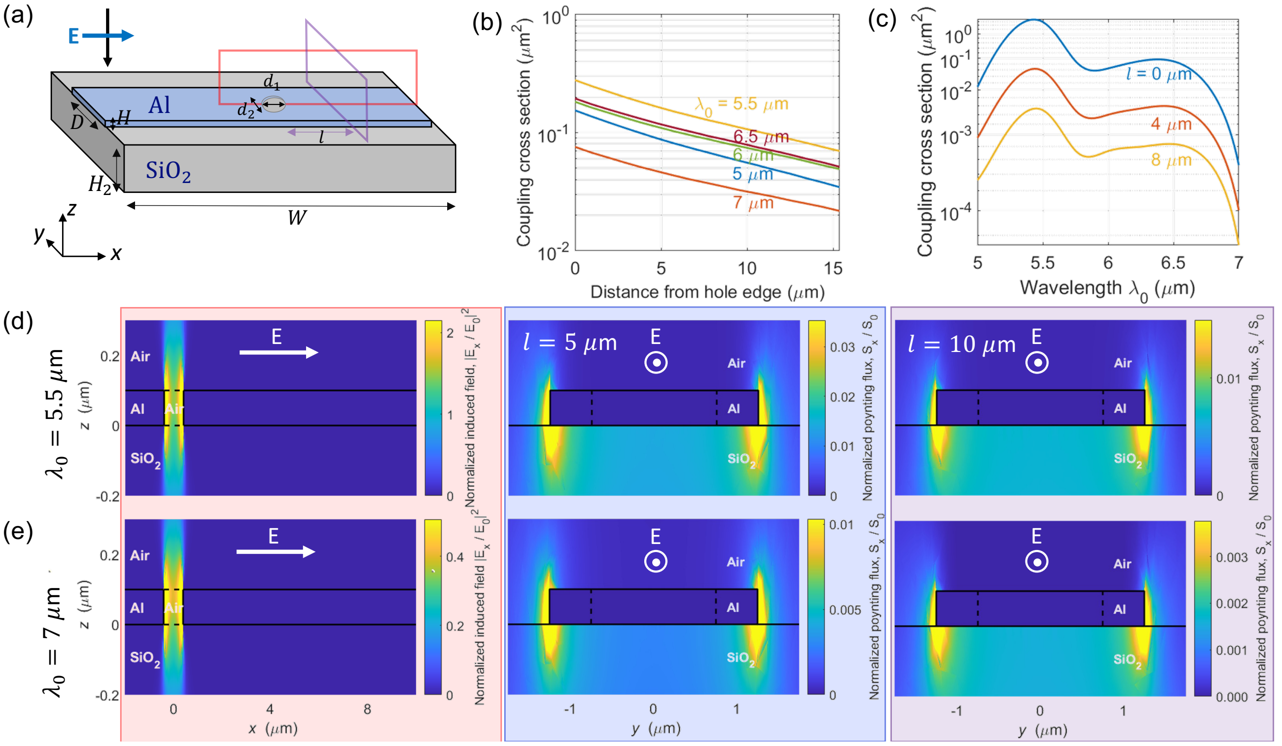

FIG. S3: **Coupling from external light to guided plasmons through an elliptical aperture.** **(a)** We consider an aluminum waveguide perforated by an elliptical hole and illuminated by a normally incident light plane wave polarized along x (i.e., along the waveguide direction). **(b)** Coupling cross section as a function of distance l along the waveguide relative to the rightmost edge of the hole. The cross section is obtained for selected values of the light wavelength λ_0 by integrating the x component of the Poynting vector over a transverse plane and dividing the result by the incident plane-wave intensity. **(c)** Same as (b), as a function of light wavelength λ_0 for selected values of the distance l. **(d)** Near-field and Poynting vector maps for a light wavelength $\lambda_0 = 5.5$ μm. Left: induced near-electric-field intensity within the longitudinal color-matched rectangle shown in (a) (in the $x - z$ plane). Middle and right: spatial distribution of the Poynting vector component parallel to x (waveguide direction) within the transverse color-matched rectangle in (a) ($y - z$ plane) at distances $l = 5$ μm and 10 μm, respectively. **(e)** Same as (c), but for a light wavelength $\lambda_0 = 7$ μm. In all calculations, we consider a silica-supported aluminum waveguide of width $D = 2.5$ μm, thickness $H = 100$ nm, and total length $W = 25$ μm, perforated by an aperture of diameters $d_1 = 0.8$ μm and $d_2 = 1.5$ μm, as shown in (a).

where we separate in- and out-of-plane components relative to the aluminum-silicon interface with energies $\hbar\varepsilon_Q \equiv \hbar^2 Q^2/2m_e$ and $\hbar\varepsilon_n$, respectively, $\mathbf{Q} = (Q_y, Q_z)$ is the 2D in-plane wave vector, the index n runs over out-of-plane states, $\mathbf{R} = (y, z)$ are in-plane real coordinates, and A is a quantization area. The process under study is linear in the external field intensity, so the light-electron interaction can be introduced through the linear term of the minimal coupling Hamiltonian,

$$\hat{\mathcal{H}}_1(\mathbf{r}, t) = -\frac{\hbar e}{m_e \omega}\, \mathrm{e}^{-\mathrm{i}\omega t}\, \mathbf{E}(\mathbf{r}) \cdot \nabla + \mathrm{c.c.}, \tag{S2}$$

where we work in a gauge with vanishing scalar potential and take $\nabla \cdot \mathbf{E}(\mathbf{r}) = 0$. The latter is a rigorous choice inside a homogeneous medium, and we assume it also applies at the interface.

To first order in the interaction, the perturbed wave function is given by

$$\psi^1_{\mathbf{Q},n}(\mathbf{r}, t) = \int d^3\mathbf{r}' \int dt'\, G_0(\mathbf{r}, \mathbf{r}', t - t')\, \hat{\mathcal{H}}_1(\mathbf{r}', t')\, \psi^0_{\mathbf{Q},n}(\mathbf{r}', t'), \tag{S3}$$

where we introduce the unperturbed Green function implicitly defined by $(\hat{\mathcal{H}}_0 - \mathrm{i}\hbar\partial_t) G_0(\mathbf{r}, \mathbf{r}', t - t') = -\delta(\mathbf{r} - \mathbf{r}')\delta(t - t')$. Invoking the in-plane translational invariance of the system, it is convenient to write the Green function as

$$G_0(\mathbf{r}, \mathbf{r}', t - t') = \int \frac{d^2\mathbf{Q}}{(2\pi)^2} \int \frac{d\varepsilon}{2\pi}\, \mathrm{e}^{\mathrm{i}\mathbf{Q}\cdot(\mathbf{R}-\mathbf{R}')}\, \mathrm{e}^{-\mathrm{i}(\varepsilon+\varepsilon_Q)(t-t')}\, G_0(x, x', \varepsilon) \tag{S4}$$

in terms of components $G_0(x, x', \varepsilon)$ defined by the equation $(\hat{\mathcal{H}}_0 - \hbar\varepsilon) G_0(x, x', \varepsilon) = -\delta(x - x')$ and associated with specific values of the out-of-plane energy $\hbar\varepsilon$ (see Appendix A).

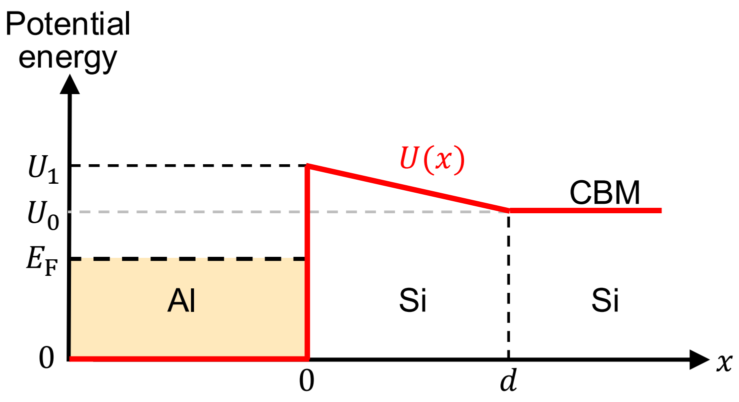

FIG. S4: **Electron potential energy landscape.** We model the evolution of electrons excited from aluminum and transmitted to silicon through an electron Green function constructed from the potential energy landscape $U(x)$ plotted in this figure. We identify $U(x)$ with the conduction band minimum (CBM) as a function of distance x relative to the interface (lying in the $x=0$ plane). Conduction electrons in aluminum span a band limited by the Fermi energy E_F. The CBM in bulk silicon lies at an energy U_0 relative to the CBM in aluminum. At the interface, there is a potential energy barrier where the silicon CBM reaches U_1 and is assumed to decay linearly down to U_0 over a distance d. Note that the actual barrier has an exponential-like profile near the interface, and thus, our calculated current constitutes a conservative estimate. In addition, the parameters d and U_1-U_0 have to be considered as effective. For simplicity, we take the effective electron mass as the free-electron mass m_e throughout the structure. This is a good approximation for aluminum, a paradigmatic free-electron metal with $E_F=(\hbar^2/2m_e)(3\pi^2 n)^{2/3}=11.6\,$eV based on the electron density $n=12/a^3$ corresponding to three electrons per atom and an fcc lattice constant $a=0.405\,$nm. We ignore effects associated with the complex band structure of silicon, including the fact that it has six CBMs and an anisotropic effective mass.

Introducing Eqs. (S1), (S2), and (S4) into Eq. (S3), and retaining only the contribution that increases the electron energy by $\hbar\omega$ (since we are interested in plasmon-assisted tunneling), the first-order perturbation of the wave function reduces to

$$\psi^1_{\mathbf{Q},n}(\mathbf{r},t)=A^{-1/2}\mathrm{e}^{\mathrm{i}\mathbf{Q}\cdot\mathbf{R}}\,\varphi^1_n(x)\,\mathrm{e}^{-\mathrm{i}(\varepsilon_n+\omega+\varepsilon_Q)t}, \tag{S5}$$

where

$$\varphi^1_n(x)=-\frac{\hbar e}{m_e\omega}\int dx'\,G_0(x,x',\varepsilon_n+\omega)\,E_x(x')\,\partial_{x'}\varphi^0_n(x') \tag{S6}$$

represents the out-of-plane component at the final out-of-plane energy $\hbar(\varepsilon_n+\omega)$. Here, we have used the fact that the final out-of-plane state is orthogonal to the initial one, so only the x component of the electric field produces a nonvanishing contribution. In addition, we have neglected the dependence of the field on in-plane coordinates $\mathbf{R}=(y,z)$, as the associated optical wave vectors are small compared with the electron wave vectors over the integration area in $\mathbf{Q}$ (see below).

Finally, the electron tunneling current density (i.e., number of electrons per unit time and transverse area) can be calculated from the perturbation wave function in Eq. (S5) as

$$\begin{aligned}J&=\frac{2\hbar}{m_e}\sum_n\sum_{\mathbf{Q}}\Theta[E_F-\hbar(\varepsilon_n+\varepsilon_Q)]\,\Theta[\hbar(\varepsilon_n+\omega+\varepsilon_Q)-U_0]\,\mathrm{Im}\{\psi^{1*}_{\mathbf{Q},n}(\mathbf{r},t)\partial_x\psi^1_{\mathbf{Q},n}(\mathbf{r},t)\}\\&=\frac{\hbar}{\pi m_e}\sum_n\int_0^\infty QdQ\,\Theta[E_F-\hbar(\varepsilon_n+\varepsilon_Q)]\,\Theta[\hbar(\varepsilon_n+\omega+\varepsilon_Q)-U_0]\,\mathrm{Im}\{\varphi^{1*}_n(x)\partial_x\varphi^1_n(x)\},\end{aligned} \tag{S7}$$

where the sum over initial states is restricted by the conditions that the initial energy lies below E_F (i.e., occupied states in aluminum, assuming zero temperature) and the final state is above U_0 (i.e., in the conduction band of bulk silicon). Note that we have introduced an overall factor of 2 to account for spin degeneracy and made the substitution $\sum_{\mathbf{Q}}\to A\int d^2\mathbf{Q}/(2\pi)^2\to A\int_0^\infty QdQ/2\pi$ to derive the second line of Eq. (S7).

The current needs to be evaluated at a sufficiently large distance x inside bulk silicon, where the incident electron wave function (which is evanescent outside aluminum) takes negligible values. In addition, we have

$$G_0(x,x',\varepsilon)=\mathrm{e}^{\mathrm{i}q'x}g_0(x',\varepsilon) \tag{S8}$$

for $x>d,x'$ (see Appendix A), corresponding to rightward electron propagation with a wave vector $q'=$

$\hbar^{-1}\sqrt{2m_e(\hbar\varepsilon - U_0)}$. Using Eqs. (S6) and (S8) to manipulate Eq. (S7), the electron current density reduces to

$$J = \frac{\hbar^3 e^2}{\pi m_e^3 \omega^2} \sum_n \int_0^\infty dQ\, q'_\omega Q\, \Theta[E_F - \hbar(\varepsilon_n + \varepsilon_Q)]\, \Theta[\hbar(\varepsilon_n + \omega + \varepsilon_Q) - U_0] \times \left| \int dx\, g_0(x, \varepsilon_n + \omega)\, E_x(x)\, \partial_x \varphi_n^0(x) \right|^2, \tag{S9}$$

where $q'_\omega = \hbar^{-1}\sqrt{2m_e[\hbar(\varepsilon + \omega) - U_0]}$. To deal with the sum over n (the index of out-of-plane unperturbed wave functions), we consider the metal to be flanked by an infinite potential barrier at $x = -L$, such that discrete solutions are obtained for $\varphi_n^0(x)$ corresponding to wave vectors $q_n = (n\pi + \phi)/L$, where ϕ is a phase associated with electron reflection at the aluminum-silicon interface (see Appendix B). In the $L \to \infty$ limit, we can transform the sum into an integral over wave vectors according to $\sum_n \to (L/\pi) \int_0^\infty dq$. Now, using Eq. (S1) (Appendix B), the L factor drops and Eq. (S9) becomes

$$J = \frac{\hbar^3 e^2}{2\pi^2 m_e^3 \omega^2}\, \Theta(E_F + \hbar\omega - U_0) \int_{k_\omega}^{k_F} dq \int_0^{\sqrt{{k_F}^2 - q^2}} dQ\, q'_\omega Q \left| \int dx\, g_0(x, \varepsilon + \omega)\, E_x(x)\, \partial_x \psi_q(x) \right|^2, \tag{S10}$$

where $\varepsilon = \hbar q^2/2m_e$ is expressed in terms of the electron wave vector inside the metal, $q = \sqrt{2m_e\varepsilon/\hbar}$; the former step functions are incorporated in the integration limits through $k_F = \sqrt{2m_e E_F}/\hbar$ and $k_\omega = \sqrt{2m_e(U_0 - \hbar\omega)}/\hbar$; and the functions $g_0(x, \varepsilon)$ and $\psi_q(x)$ are defined in Eqs. (S2) and (S2), respectively. Obviously, the current vanishes if $E_F + \hbar\omega < U_0$ (i.e., when aluminum conduction electrons cannot be promoted above U_0, the CBM of bulk silicon).

S3. RELATION BETWEEN TUNNELING CURRENT AND OPTICAL INTENSITY AT THE INTERFACE

In the analysis presented in Sec. S2, we ignore the dependence of the optical field on the coordinates $\mathbf{R} = (y, z)$ within the transverse cross section of the aluminum waveguide. This is a reasonable approximation if we consider that such a field varies over characteristic distances of a few nanometers (even at the edge of the waveguide), which are large compared to the involved electron wavelengths, so that tunneling occurs locally at each $\mathbf{R}$ position, and transverse optical wave vectors can be ignored.

To obtain an estimate of the linear relation between the tunneling current and the field intensity, we consider that tunneling electrons originate from the excitation of aluminum conduction electrons at a small distance from the interface with silicon, as dictated by the inelastic electron mean free path, which we approximate to an effective value $L_i^{\rm eff} = 10$ nm. Although we cannot find experimental measurements for this parameter in crystalline aluminum for electron energy at < 1 eV above the Fermi level, this value is consistent with available *ab initio* calculations [3, 4]. We also assume the optical electric field to vary negligibly over such a distance and approximate the interface-normal component as $E_x(\mathbf{r}) \approx E_x^-(\mathbf{R})\Theta(-x) + E_x^+(\mathbf{R})\Theta(x)$, where the functions $E_x^\pm(\mathbf{R})$ depend on the coordinates $\mathbf{R} = (y, z)$ parallel to the interface. Because of the continuity of the normal electric displacement, we have $E_x^+(\mathbf{R}) = E_x^-(\mathbf{R})\eta$, where $\eta = \epsilon_{\rm Al}(\omega)/\epsilon_{\rm Si}(\omega)$ is the ratio of permittivities in the two materials. These expressions describe a large jump in the normal electric field at the interface because $|\eta| \gg 1$. Introducing these approximations in Eq. (S10) and integrating over the interface area A (i.e., the cross section at the end of the aluminum waveguide), the electron current (electrons per unit time) reduces to

$$I_e = \frac{\alpha\hbar^4 I_0}{\pi m_e^3 \omega^2}\, \Theta(E_F + \hbar\omega - U_0)\, A^- \int_{k_\omega}^{k_F} dq \int_0^{\sqrt{{k_F}^2 - q^2}} dQ\, q'_\omega Q \left[\left|F^-\right|^2 + |\eta|^2 \left|F^+\right|^2 + 2{\rm Re}\{\eta F^+ F^{-*}\} \right], \tag{S11}$$

where

$$A^- = \frac{1}{E_0^2} \int_A d^2\mathbf{R}\, \left|E_x^-(\mathbf{R})\right|^2,$$
$$F^- = \int_{-\infty}^0 dx\, g_0(x, \varepsilon + \omega)\, \partial_x \psi_q(x),$$
$$F^+ = \int_0^\infty dx\, g_0(x, \varepsilon + \omega)\, \partial_x \psi_q(x),$$

$I_0 = (c/2\pi)E_0^2$ is the incident light intensity (power per unit area) corresponding to the incident field amplitude E_0, and $\alpha = e^2/\hbar c \approx 1/137$ is the fine structure constant.

As noted above, the metal region in which internal photoemission contributes to tunneling is limited to a distance $L_i^{\rm eff}$ from the interface due to inelastic electron collisions. We incorporate this effect directly through

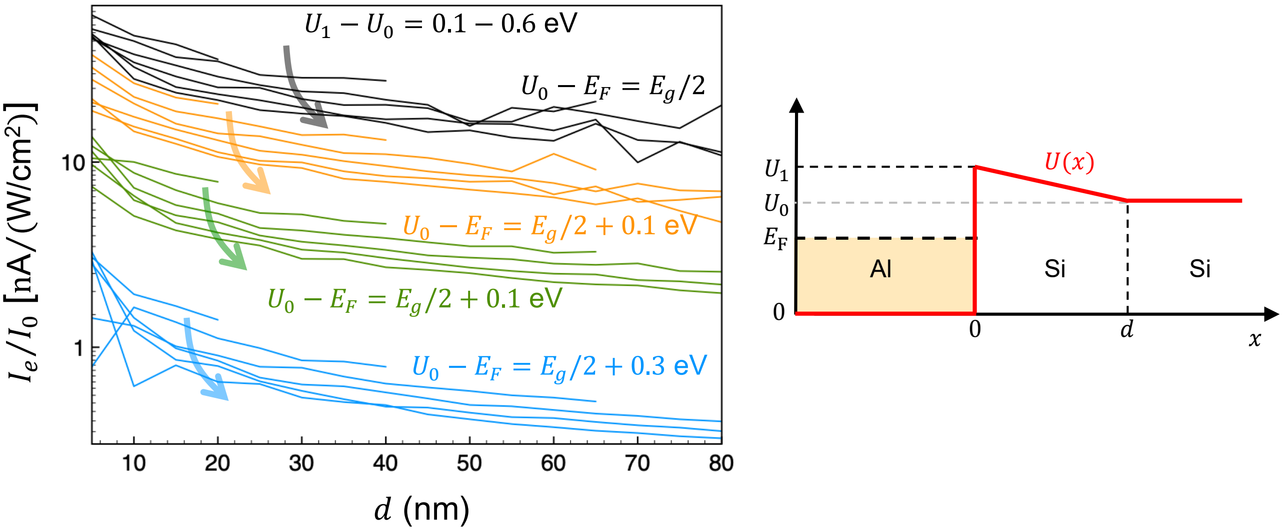

FIG. S5: **Calculated photon-assisted tunneling current.** We plot the tunneling current I_e (nA) normalized to the incident light intensity I_0 (W/cm^2) as calculated from Eq. (S11) for a light wavelength of 1.3 μm, assuming the electron potential energy landscape of Fig. S4 (reproduced in the right part of this figure for the sake of readability). The current is represented as a function of barrier width d for different values of $U_1 - U_0$ and $U_0 - E_F$, as indicated by labels. In particular, $U_0 - E_F$ is expressed in terms of the silicon gap energy $E_g = 1.1$ eV.

an imaginary part in the electron wave vector inside aluminum (see Appendix A). With this correction, we use Eq. (S11) to estimate the electron current produced for a given incident electron intensity. From the electromagnetic calculations in Sec. S1, considering a light wavelength $\lambda_0 = 1.3\,\mu$m, we find $A^- = 1670$ nm^2 when integrated over the 2.5 μm$\times$100 nm area of the aluminum-silicon interface. Direct integration of Eq. (S11) with this parameter leads to the result plotted in Fig. S5, showing an expected attenuation of I_e/I_0 with the width and relative height of the barrier (d and $U_1 - U_0$, respectively), as well as with the jump from E_F to U_0. In the absence of a back gate, the latter can be estimated as $U_0 - E_F = E_g/2 = 0.55$ eV from the silicon gap energy E_g. We also show results for higher barriers $U_0 - E_F$, increased by $0.1 - 0.3$ eV.

Appendix A: Electron Green function

The Green function $G_0(x, x', \varepsilon)$ defined in Sec. S2 satisfies the relation $(\hat{\mathcal{H}}_0 - \hbar\varepsilon)G_0(x, x', \varepsilon) = -\delta(x - x')$. We solve this equation for each fixed value of x' by considering the homogeneous solutions of $(\hat{\mathcal{H}}_0 - \hbar\varepsilon)\varphi(x) = 0$ in different regions of the potential energy landscape (see Fig. S4). In particular, we have $\varphi(x) = \mathrm{e}^{\pm \mathrm{i}qx}$ with $q = \sqrt{2m_\mathrm{e}\varepsilon/\hbar}$ for $x < 0$ and $\varphi(x) = \mathrm{e}^{\pm \mathrm{i}q'x}$ for $x > d$ with $q' = \hbar^{-1}\sqrt{2m_\mathrm{e}(\hbar\varepsilon - U_0)}$ [see Eq. (S8)], while $\varphi(x)$ admits the solutions

$$\chi_1(x) = \mathrm{Ai}(\theta),$$
$$\chi_2(x) = \mathrm{Bi}(\theta)$$

in the $0 < x < d$ region, where Ai and Bi are Airy functions [5] evaluated at $\theta = -(2m_\mathrm{e}a/\hbar^2)^{1/3}\left[x + (\hbar\varepsilon - U_0)/a\right]$ with $a = (U_1 - U_0)/d$. In addition, we impose the continuity of $G_0(x, x', \varepsilon)$ everywhere as a function of x (in particular at $x = 0$, d, and x'), as well as the continuity of $\partial_x G_0(x, x', \varepsilon)$ (in particular at $x = 0$ and d), except at $x = x'$, where it presents and a jump

$$\partial_x G_0(x, x', \varepsilon)\big|_{x=x'+0^+} - \partial_x G_0(x, x', \varepsilon)\big|_{x=x'-0^+} = \frac{2m_\mathrm{e}}{\hbar^2}$$

to account for the $\delta(x - x')$ function in the above expression.

Focusing on the retarded solution of the Green function behaving as $\propto \mathrm{e}^{-\mathrm{i}qx}$ at $x \to -\infty$ and $\propto \mathrm{e}^{\mathrm{i}q'x}$ at

$x \to \infty$, we can write

$$G_0(x,x',\varepsilon) = \frac{2m_\mathrm{e}}{\hbar^2} \times \begin{cases} \left.\begin{array}{ll} A_1(x')\,\mathrm{e}^{-\mathrm{i}qx}, & x<x' \\ B_1(x')\,\mathrm{e}^{\mathrm{i}qx} + C_1(x')\,\mathrm{e}^{-\mathrm{i}qx}, & x'<x<0 \\ D_1(x')\,\chi_1(x) + E_1(x')\,\chi_2(x), & 0<x<d \\ F_1(x')\,\mathrm{e}^{\mathrm{i}q'x}, & d<x \end{array}\right\} & x'<0, \\ \\ \left.\begin{array}{ll} A_2(x')\,\mathrm{e}^{-\mathrm{i}qx}, & x<0 \\ B_2(x')\,\chi_1(x) + C_2(x')\,\chi_2(x), & 0<x<x' \\ D_2(x')\,\chi_1(x) + E_2(x')\,\chi_2(x), & x'<x<d \\ F_2(x')\,\mathrm{e}^{\mathrm{i}q'x}, & d<x \end{array}\right\} & 0<x'<d, \\ \\ \left.\begin{array}{ll} A_3(x')\,\mathrm{e}^{-\mathrm{i}qx}, & x<0 \\ B_3(x')\,\chi_1(x) + C_3(x')\,\chi_2(x), & 0<x<d \\ D_3(x')\,\mathrm{e}^{\mathrm{i}q'x} + E_3(x')\,\mathrm{e}^{-\mathrm{i}q'x}, & d<x<x' \\ F_3(x')\,\mathrm{e}^{\mathrm{i}q'x}, & x'<x \end{array}\right\} & d<x', \end{cases}$$

where we separately obtain the solutions in the three indicated x' regions in terms of x'-dependent coefficients, which bear an implicit dependence on ε through q and q'. From these expressions, by applying the noted boundary conditions, we obtain the matrix equations

$$\begin{bmatrix} \mathrm{e}^{-\mathrm{i}qx'} & -\mathrm{e}^{\mathrm{i}qx'} & -\mathrm{e}^{-\mathrm{i}qx'} & 0 & 0 & 0 \\ -\mathrm{i}q\mathrm{e}^{-\mathrm{i}qx'} & -\mathrm{i}q\mathrm{e}^{\mathrm{i}qx'} & \mathrm{i}q\mathrm{e}^{-\mathrm{i}qx'} & 0 & 0 & 0 \\ 0 & 1 & 1 & -\chi_1(0) & -\chi_2(0) & 0 \\ 0 & \mathrm{i}q & -\mathrm{i}q & -\chi_1'(0) & -\chi_2'(0) & 0 \\ 0 & 0 & 0 & \chi_1(d) & \chi_2(d) & -\mathrm{e}^{\mathrm{i}q'd} \\ 0 & 0 & 0 & \chi_1'(d) & \chi_2'(d) & -\mathrm{i}q'\mathrm{e}^{\mathrm{i}q'd} \end{bmatrix} \cdot \begin{bmatrix} A_1(x') \\ B_1(x') \\ C_1(x') \\ D_1(x') \\ E_1(x') \\ F_1(x') \end{bmatrix} = \begin{bmatrix} 0 \\ 1 \\ 0 \\ 1 \\ 0 \\ 1 \end{bmatrix}, \tag{S1a}$$

$$\begin{bmatrix} 1 & -\chi_1(0) & -\chi_2(0) & 0 & 0 & 0 \\ -\mathrm{i}q & -\chi_1'(0) & -\chi_2'(0) & 0 & 0 & 0 \\ 0 & \chi_1(x') & \chi_2(x') & -\chi_1(x') & -\chi_2(x') & 0 \\ 0 & \chi_1'(x') & \chi_2'(x') & -\chi_1'(x') & -\chi_2'(x') & 0 \\ 0 & 0 & 0 & \chi_1(d) & \chi_2(d) & -\mathrm{e}^{\mathrm{i}q'd} \\ 0 & 0 & 0 & \chi_1'(d) & \chi_2'(d) & -\mathrm{i}q'\mathrm{e}^{\mathrm{i}q'd} \end{bmatrix} \cdot \begin{bmatrix} A_2(x') \\ B_2(x') \\ C_2(x') \\ D_2(x') \\ E_2(x') \\ F_2(x') \end{bmatrix} = \begin{bmatrix} 0 \\ 1 \\ 0 \\ 1 \\ 0 \\ 1 \end{bmatrix}, \tag{S1b}$$

$$\begin{bmatrix} 1 & -\chi_1(0) & -\chi_2(0) & 0 & 0 & 0 \\ -\mathrm{i}q & -\chi_1'(0) & -\chi_2'(0) & 0 & 0 & 0 \\ 0 & \chi_1(d) & \chi_2(d) & -\mathrm{e}^{\mathrm{i}q'd} & -\mathrm{e}^{-\mathrm{i}q'd} & 0 \\ 0 & \chi_1'(d) & \chi_2'(d) & -\mathrm{i}q'\mathrm{e}^{\mathrm{i}q'd} & \mathrm{i}q'\mathrm{e}^{-\mathrm{i}q'd} & 0 \\ 0 & 0 & 0 & \mathrm{e}^{\mathrm{i}q'x'} & \mathrm{e}^{-\mathrm{i}q'x'} & -\mathrm{e}^{\mathrm{i}q'x'} \\ 0 & 0 & 0 & \mathrm{i}q'\mathrm{e}^{\mathrm{i}q'x'} & -\mathrm{i}q'\mathrm{e}^{-\mathrm{i}q'x'} & -\mathrm{i}q'\mathrm{e}^{\mathrm{i}q'x'} \end{bmatrix} \cdot \begin{bmatrix} A_3(x') \\ B_3(x') \\ C_3(x') \\ D_3(x') \\ E_3(x') \\ F_3(x') \end{bmatrix} = \begin{bmatrix} 0 \\ 1 \\ 0 \\ 1 \\ 0 \\ 1 \end{bmatrix}. \tag{S1c}$$

Finally, the function $g_0(x',\varepsilon)$ defined in Eq. (S8) is given by

$$g_0(x',\varepsilon) = \frac{2m_\mathrm{e}}{\hbar^2} \times \begin{cases} F_1(x'), & x'<0, \\ F_2(x'), & 0<x'<d, \\ F_3(x'), & d<x', \end{cases} \tag{S2}$$

where the $F_j(x')$ coefficients are obtained by solving Eqs. (S1). This leads to

$$g_0(x',\varepsilon) = -\frac{2m_\mathrm{e}}{\hbar^2} \times \frac{(1+\mathrm{e}^{-\mathrm{i}qx'})[\chi_1'(d)\chi_2(d) - \chi_1(d)\chi_2'(d)] - \{\chi_1(d)[\chi_2'(0)+\mathrm{i}q\chi_2(0)] - [\chi_1'(0)+\mathrm{i}q\chi_1(0)]\chi_2(d)\}}{[\chi_1'(d) - \mathrm{i}q'\chi_1(d)][\chi_2'(0)+\mathrm{i}q\chi_2(0)] - [\chi_1'(0)+\mathrm{i}q\chi_1(0)][\chi_2'(d) - \mathrm{i}q'\chi_2(d)]}$$

for $x'<0$, and more complex expressions for the other two x' regions, which we evaluate numerically using standard linear algebra techniques.

The above expressions yield the Green function for an electron evolving in a conservative potential. We now incorporate the effect of inelastic collisions in the metal by adding a small imaginary part to the electron wave vector in the $x<0$ region. More precisely, we perform the substitution $q \to q + \mathrm{i}/2L_\mathrm{i}^\mathrm{eff} = \sqrt{2m_\mathrm{e}\varepsilon/\hbar} + \mathrm{i}/2L_\mathrm{i}^\mathrm{eff}$, where $L_\mathrm{i}^\mathrm{eff}$ represents an effective attenuation length, while q' is still given by Eq. (**??**) (i.e., no attenuation in silicon).

Appendix B: Unperturbed electron wave functions

Conduction electrons in the aluminum gate can be indexed by the wave vector q for each energy $\hbar\varepsilon < E_F$. Considering the potential landscape of Fig. S4, the wave vector on bulk silicon becomes imaginary [$q' = \mathrm{i}\kappa$ with $\kappa = \hbar^{-1}\sqrt{2m_\mathrm{e}(U_0 - \hbar\varepsilon)}$]. Following an analysis similar to Appendix A, we can write the corresponding wave functions as

$$\varphi_q(x) = \frac{1}{\sqrt{2L}}\,\psi_q(x), \tag{S1}$$

with

$$\psi_q(x) = \begin{cases} \mathrm{e}^{\mathrm{i}qx} + r\,\mathrm{e}^{-iqx}, & x' < 0, \\ A\chi_1(x) + B\chi_2(x), & 0 < x' < d, \\ t\,\mathrm{e}^{-\kappa(x-d)}, & d < x', \end{cases} \tag{S2}$$

where r and t play the roles of reflection and transmission coefficients, A and B are additional parametrization constants, and we introduce a length L to account for normalization (see below). From the continuity of the wave function and its derivative at $x = 0$ and d, we readily find the solution

$$\begin{aligned} r &= \mathrm{e}^{-2\mathrm{i}\phi}, \\ A &= [\kappa\chi_2(d) + \chi_2'(d)]/\Delta, \\ B &= -[\kappa\chi_1(d) + \chi_1'(d)]/\Delta, \\ t &= [\chi_1(d)\chi_2'(d) - \chi_1'(d)\chi_2(d)]/\Delta, \end{aligned}$$

where $\Delta = (2q)^{-1}\big\{[q\chi_1(0) - \mathrm{i}\chi_1'(0)][\chi_2'(d) + \kappa\chi_2(d)] - [\chi_1'(d) + \kappa\chi_1(d)][q\chi_2(0) + \mathrm{i}\chi_2'(0)]\big\}$ and $\phi = \arg\{\Delta\}$. To deal with wave-function normalization, we consider the aluminum to be limited from the left by an infinite potential barrier at $x = -L$ and eventually take the $L \to \infty$ limit. With the normalization anticipated in Eq. (S1), we indeed find $\int_{-L}^{\infty} dx\,|\varphi(x)|^2 \approx 1$, where we are neglecting a small fraction of electron probability in the $x > 0$ region. This fraction vanishes for $L \to \infty$. In addition, the wave vector must take discrete values $q_n = (n\pi + \phi)/L$ with n running over positive integers. Finally, we can write the out-of-plane wave functions appearing in Eq. (S1) as $\varphi_n^0(x) \equiv \varphi_{q_n}(x)$ and the corresponding energies as $\hbar\varepsilon_n = \hbar^2 q_n^2/2m_\mathrm{e}$.

---

[1] P. G. Silvestrov and K. B. Efetov, Phys. Rev. B **77**, 155436 (2008).

[2] N. Rotenberg, M. Spasenović, T. L. Krijger, B. le Feber, F. J. García de Abajo, and L. Kuipers, Phys. Rev. Lett. **108**, 127402 (2012).

[3] W.-D. Schöne, R. Keyling, M. Bandić, , and W. Ekardt, Phys. Rev. B **60**, 8616 (1999).

[4] I. Campillo, J. M. Pitarke, A. Rubio, E. Zarate, and P. M. Echenique, Phys. Rev. Lett. **83**, 2230 (1999).

[5] M. Abramowitz and I. A. Stegun, *Handbook of Mathematical Functions* (Dover, New York, 1972).
